# Novel Flavonoid Senotherapeutics Identified by Phenotypic Drug Discovery Reduce Senescence and Improve Multiple Markers of Healthspan

**DOI:** 10.1111/acel.70736

**Published:** 2026-10-06

**Authors:** Lei Justan Zhang, Rahagir Salekeen, Carolina Soto‐Palma, Qiuming Wu, Yuanjun He, Osama Elsallabi, Bilel Bdiri, Brian Hughes, Allancer Nunes, Wandi Xu, Borui Zhang, Abdalla Mohamed, Sara J. McGowan, Luise Angelini, Ryan O'Kelly, Theodore M. Kamenecka, Laura J. Niedernhofer, Paul D. Robbins

**Affiliations:** ^1^ Masonic Institute on the Biology of Aging and Metabolism University of Minnesota Minneapolis Minnesota USA; ^2^ Department of Biochemistry, Molecular Biology and Biophysics University of Minnesota Minneapolis Minnesota USA; ^3^ Innovations in Drug Discovery Program, Department of Pharmaceutical & Biomedical Sciences, College of Pharmacy University of Georgia Athens Georgia USA; ^4^ The Herbert Wertheim UF Scripps Institute for Biomedical Innovation & Technology Jupiter Florida USA; ^5^ Department of Medicine and Aging Sciences University “G. d'Annunzio” of Chieti‐Pescara Chieti Italy

**Keywords:** aging, fisetin analog, flavonoid, healthspan, phenotypic drug discovery, senescence, senolytic, senotherapeutic, structure–activity relationship

## Abstract

Senescent cells are a key driver of aging and contribute to many age‐related diseases. Senolytics, agents that selectively eliminate senescent cells, offer a promising therapeutic strategy to treat age‐related diseases but face significant barriers to clinical translation. Fisetin, a natural flavonoid senolytic, has progressed to clinical trials but is limited by suboptimal efficacy and poor bioavailability. To address these challenges, we developed two novel fisetin‐derived flavonoid senolytics, SR29384 and SR31133, using an integrative phenotypic drug discovery approach. These compounds exhibit enhanced senolytic activity, targeting a broad range of senescent cell types, reducing tissue senescence, and improving healthspan‐associated parameters in mouse models of aging. Mechanistic investigations combining transcriptomics, machine learning, network pharmacology, and molecular docking revealed that these novel senolytics may target key molecular pathways, including PARP1, CDK2, and possibly BCL‐xL to selectively induce apoptosis in senescent cells. These findings provide novel senolytics with high translational potential for clinical applications in promoting healthy aging and treating age‐related diseases.

## Graphical abstract

Phenotypic screening and SAR‐guided drug design identified two flavonoid‐derived senolytics, SR29384 and SR31133, which selectively killed different types of senescent cells more efficiently and with greater selectivity than fisetin in culture. In aged and progeroid mice, these fisetin analogs reduced tissue senescence and improved physical function and healthspan‐related outcomes.

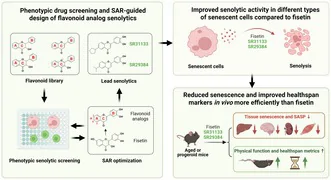

## 1 Introduction

Aging is a leading risk factor for most diseases and conditions that limit healthspan (Niccoli and Partridge 2012). As life expectancy increases, the burden of age‐related diseases on healthcare systems and economies is escalating, with trillions of dollars in annual costs projected worldwide (Jones and Dolsten 2024). This demographic shift highlights a critical need for interventions that target the root causes of aging to treat multiple diseases simultaneously (Kennedy et al. 2014). Cellular senescence, a state of stable cell cycle arrest induced by stressors such as DNA damage or oxidative stress, is emerging as one of the most fundamental root causes or hallmarks of aging (Hayflick and Moorhead 1961; Lopez‐Otin et al. 2013, 2023).

With age, senescent cells accumulate in tissues and secrete a range of deleterious signals known as the senescence‐associated secretory phenotype (SASP) (Coppe et al. 2010; Di Micco et al. 2021). The SASP comprises a complex mix of pro‐inflammatory cytokines, chemokines, proteases, nucleic acids, and metabolites that exacerbate tissue dysfunction and promote systemic inflammation. Through paracrine and endocrine signaling, these factors create a feedback loop that accelerates aging and drives the development of many age‐related diseases (Birch and Gil 2020; Gorgoulis et al. 2019; Wiley and Campisi 2021). Studies using transgenic mouse models, where *p16* ^*Ink4a*^ or *p21* ^*Cip1*^‐positive senescent cells can be selectively ablated, have demonstrated the pivotal role of senescent cells in driving aging and age‐related diseases (Baker et al. 2016, 2011; Wang et al. 2021). Moreover, pharmacological approaches targeting senescent cells using a class of drugs known as senolytics have shown promise in mitigating age‐associated dysfunction, extending healthspan, and treating many age‐related diseases (Kirkland and Tchkonia 2020; Prasnikar et al. 2021; Robbins et al. 2021).

Despite the promise, the number of effective senolytics identified remains limited. Many senolytics, including the combination of dasatinib and quercetin, navitoclax, and fisetin, were discovered through bioinformatics or focused compound screens and face challenges such as poor bioavailability, high toxicity, or limited efficacy (Tse et al. 2008; Zhang et al. 2021, 2023; Zhang et al. 2022). Among these, fisetin, a naturally occurring flavonoid, has shown potential as a senolytic in preclinical studies. Fisetin has been reported to reduce senescence markers, alleviate inflammation, suppress age‐related pathologies, and extend healthspan in aged mice (Yousefzadeh et al. 2018; Zhu et al. 2017). These findings have led to multiple clinical trials investigating fisetin for various conditions, such as chronic kidney disease (NCT03325322), skeletal health (NCT04313634), osteoarthritis (NCT04210986), COVID‐19 (NCT04476953, NCT04537299), and frailty (NCT03675724). However, fisetin is not a strong senolytic, requiring high doses in cell culture for senolysis and in animal studies to achieve therapeutic effects (Yousefzadeh et al. 2018). Furthermore, fisetin suffers from poor pharmacokinetic properties, such as low solubility and absorption, potentially limiting its clinical effectiveness (Jo et al. 2016; Seguin et al. 2013).

To overcome these limitations, we employed a phenotypic drug discovery approach to develop optimized senolytics derived from fisetin. Through structure–activity relationship (SAR) analyses and preclinical testing, we successfully developed two novel flavonoid senolytics, SR29384 and SR31133. Preclinical investigation demonstrated that these novel flavonoid senolytics can efficiently reduce tissue senescence and improve healthspan‐associated markers in aging mouse models. Comprehensive mechanistic studies revealed that these compounds modulate key molecular targets, such as PARP1 and CDK2, to selectively induce apoptosis in senescent cells. As such, these novel compounds represent promising senotherapeutic candidates for the clinical translation of senescence‐targeting therapies to target age‐related diseases.

## 2 Results

### 2.1 Phenotypic Drug Screening of Natural Flavonoids for Potent Senolytics

Flavonoids are a diverse class of polyphenolic natural products found in various fruits, vegetables, and plants. These compounds are characterized by a diphenylpropane scaffold, commonly referred to as a C_6_‐C_3_‐C_6_ structural framework, and are categorized into several subgroups based on their chemical structures, including flavones, isoflavones, flavonols, flavanols, and anthocyanidins. Flavonoids have been extensively studied for their wide range of biological activities, such as antioxidant, anti‐inflammatory, antidiabetic, antimicrobial, antimalarial, antiviral, anticancer, and neuroprotective effects (Proshkina et al. 2024). As a member of this family, fisetin also exhibits diverse activities, including senolytic properties (Yousefzadeh et al. 2018), which extend beyond the common activities of flavonoids (Rahmani et al. 2022; Ravula et al. 2021; Sundarraj et al. 2018). However, fisetin is known for its promiscuity in targeting multiple pathways, which makes its molecular mechanisms complex (Rahmani et al. 2022; Ravula et al. 2021; Sundarraj et al. 2018). For example, fisetin can activate or suppress various molecular targets and pathways, enhancing antioxidant status, inducing apoptosis, and inhibiting proliferation, inflammation, and angiogenesis. Due to these complexities, traditional structure‐based drug design approaches, which rely on targeting a predefined protein or biomolecule, are not well‐suited for developing novel senolytic flavonoid analogs (FAs). To overcome this challenge, we employed a phenotypic drug discovery approach, leveraging senescent cell‐based screening to evaluate various flavonoids for their senolytic activity.

We previously developed an efficient senotherapeutic screening platform based on senescence‐associated beta‐galactosidase (SA‐β‐gal) activity as a primary marker and phenotype of senescent cells (Gorgoulis et al. 2019; Zhang et al. 2022). This platform, equipped with a high‐content fluorescent imaging system, allows quick evaluation of both senolytics and senomorphics. Using this platform, we screened a focused library of various natural flavonoids, including flavones, flavonols, flavanols, anthocyanidins, and other subclasses, based on their ability to modulate the phenotype of SA‐β‐gal activity (Figure 1A, and Figure S1).

**FIGURE 1:**
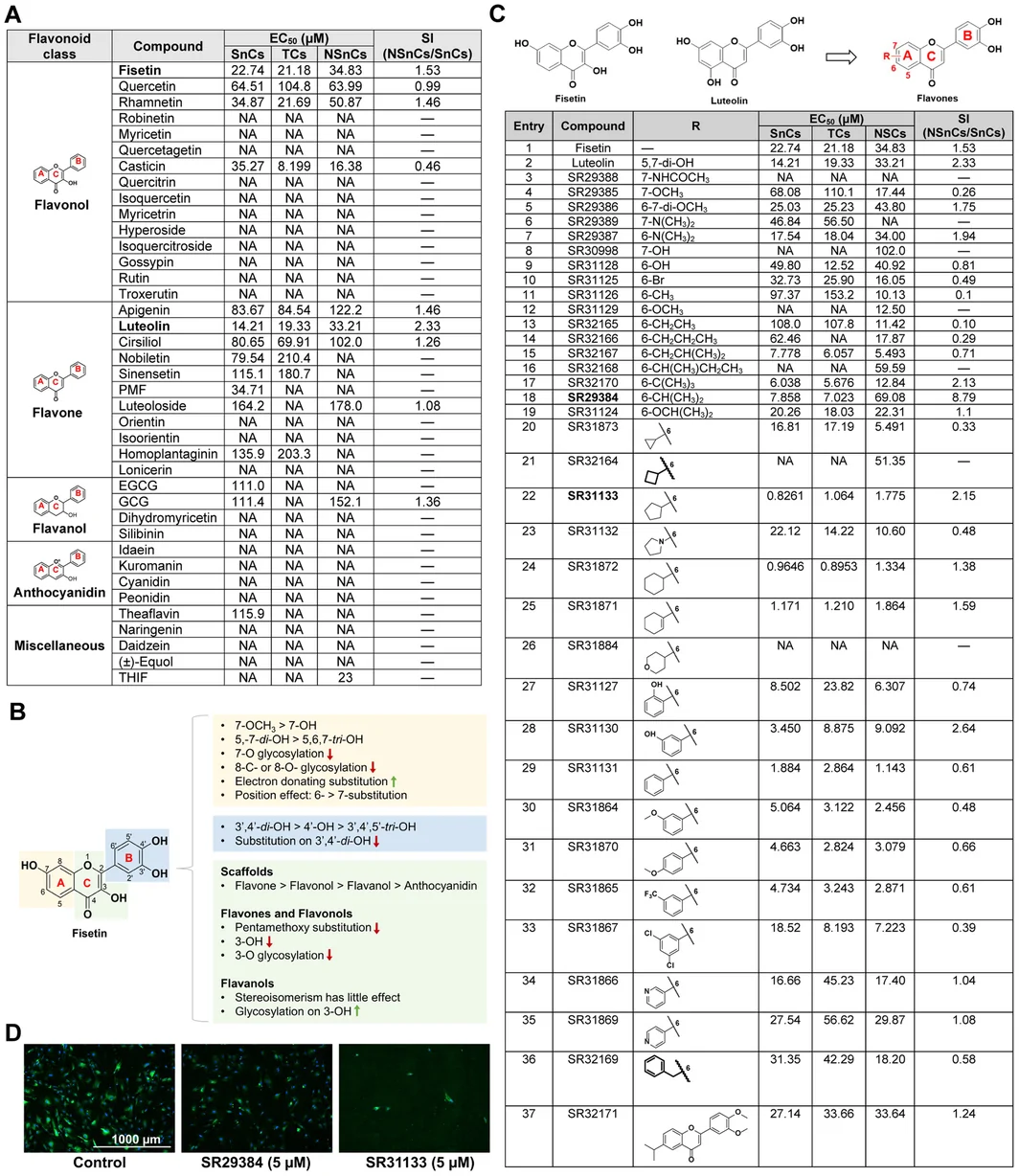
Discovery of novel flavonoid senolytics by phenotypic drug discovery. (A) Summary of the preliminary senescence screening of flavonoid analogs of fisetin in senescent *Ercc1* ^−/−^ and non‐senescent WT MEFs. (B) Summary of the preliminary structure–activity relationship from the phenotypic drug screening of natural flavonoids. (C) Summary of the structural optimization for improved flavonoid analogs. Green upward arrows indicate structural modifications that enhance senolytic potency, whereas red downward arrows indicate modifications that reduce potency. (D) Representative images of the senescence assay showing SR29384 and SR31133 reduced SA‐β‐gal positive senescent *Ercc1* ^−/−^ MEF cells after 48 h of treatment. Blue fluorescence indicates nuclei stained with Hoechst 33342, and green fluorescence indicates SA‐β‐gal positive senescent cells stained with C_12_FDG. Images were captured using Cytation 1 at 4× magnification. Abbreviations: SnCs: Senescent *Ercc1* ^−/−^ MEF cells; TCs: Total *Ercc1* ^−/−^ MEF cells; NSnCs: Non‐senescent WT MEF cells; SI: Selectivity index; EC_50_: The concentration that results in a 50% decrease in the number of cells compared with that of the control. NA: EC_50_ values are not available due to inactivity. PMF: 3′,4′,5′,5,7‐pentamethoxyflavone; EGCG: (−)‐epigallocatechin gallate; GCG: (−)‐gallocatechin gallate; THIF: 3′,4′,7‐trihydroxyisoflavone.

The senolytic activity of these compounds was assessed initially in *Ercc1* ^−/−^ senescent mouse embryonic fibroblasts (MEFs). The ERCC1 protein is a subunit of the heterodimeric ERCC1/XPF DNA repair endonuclease essential for repairing multiple types of DNA damage. *Ercc1* ^−/Δ^ mice, with reduced expression of ERCC1, show increased sensitivity to DNA damage, elevated senescent cell burden, accelerated aging, and a shortened lifespan (Gurkar and Niedernhofer 2015; Yousefzadeh et al. 2020). Accordingly, primary *Ercc1* ^−/−^ MEFs with impaired DNA damage repair readily become senescent under oxidative stress when passaged at 20% O_2_. The selectivity of each compound was determined using a selectivity index (SI), which compares the EC_50_ values in non‐senescent proliferating MEFs to those in senescent *Ercc1* ^−/−^ MEFs. The screening results showed that fisetin had a moderate senolytic EC_50_ value of approximately 22.74 μM and a low SI of 1.53 (Figure 1A), indicating the need for further optimization. Among the screened compounds, luteolin emerged as the most potent and selective natural flavonoid, at least in MEFs, with a senolytic EC_50_ value of 14.21 μM and an SI of 2.33 (Figure 1A).

### 2.2 Structure–Activity Relationship Analysis Guides Flavonoid Optimization

This phenotype‐based senolytic screening provided preliminary structure–activity relationship (SAR) data, which are essential for the rational design of more potent and selective flavonoid senolytics. The majority of the flavonoids tested in this study share a common structural framework, consisting of two aromatic rings A and B linked by a central three‐carbon ring C (Figure 1A and Figure S1). Based on the phenotypic screening results, we conducted a detailed SAR analysis and established fundamental SAR guidelines for each moiety of the flavonoid structure (Figure 1B). Quantitative activity and selectivity metrics used for SAR are summarized in Figure 1A–C, including senolytic EC_50_ values in senescent *Ercc1* ^−/−^ MEFs, counter‐screen EC_50_ values in non‐senescent WT MEFs, and the corresponding selectivity index (SI). The full set of tested natural flavonoids and their structures is provided in Figure S1.

#### 2.2.1 Scaffold

Across the natural flavonoids tested, the most consistent senolytic activity was observed for planar flavonoid scaffolds that retain both the C2‐C3 double bond and the C4‐oxo group (i.e., flavones and flavonols), whereas scaffolds lacking this conjugation (e.g., flavanols) or possessing alternative core chemistries (e.g., anthocyanidins, isoflavones/isoflavanes, and flavanones) were largely inactive under the same conditions (Figure 1A,B and Figure S1). Within the active scaffolds, absence of the 3‐hydroxyl group on the C ring tended to improve senolytic performance, as exemplified by luteolin (flavone; no 3‐OH) compared with quercetin (flavonol; 3‐OH). Compounds with other scaffolds, including isoflavones (daidzein and THIF), isoflavanes (equol), and flavanones (naringenin), were not effective as senolytics, further supporting the importance of the C2‐C3 double bond and the C4‐oxo group in the C ring for senolytic activity (Figure 1A,B and Figure S1).

#### 2.2.2 Ring A

The SAR analysis revealed that the presence of a 5‐hydroxyl group in the A ring decreased senolytic activity, as seen in the less potent quercetin compared to fisetin (Figure 1B and Figure S1). Electron‐donating substituents, such as methoxy groups in the A ring, were found to be favorable, evidenced by the increased potency of rhamnetin compared to quercetin. However, excessive methoxy substitutions, such as pentamethoxy groups, led to compound precipitation in cell culture media and reduced activity, as observed in nobiletin, sinensetin, and PMF. Quercetin outperformed quercetagetin, indicating that the presence of hydroxyl groups at positions 5 and 7 in the A ring is more favorable than three hydroxyl groups at positions 3, 5, and 7 in flavonols. Any glycosylation in the A ring diminished senolytic activity, as seen with gossypin compared to quercetin. This pattern was also observed for flavones. For example, glycosylation on the 7‐OH of luteolin, as in luteoloside, substantially reduced senolytic activity compared to luteolin, the most potent flavonoid in the screening. Similarly, disaccharide glycosylation on 7‐OH (lonicerin), 6‐C‐glycosylation (isoorientin), or 8‐C‐glycosylation (orientin) in the A ring of the flavone luteolin was detrimental to senolytic activity.

#### 2.2.3 Ring B

The number, position, and substitution of hydroxyl groups in the B ring are crucial for senolytic activity. This suggests that the presence of the 3′,4′‐dihydroxyl groups in the B ring is essential (Figure 1B and Figure S1), as having only one (as in apigenin < luteolin) or three (as in myricetin < quercetin) hydroxyl groups was less desirable. Although not definitive, the data also suggest that substitution on the 3′,4′‐dihydroxyl groups in the B ring might decrease activity, as observed with troxerutin.

#### 2.2.4 Ring C

Consistent with the scaffold trend, the presence of the 3‐hydroxyl group in the C ring was found to be unfavorable for senolytic activity. This was demonstrated by the reduced activity of the flavonol quercetin compared with flavone luteolin. Further SAR analysis of flavonols revealed that glycosylation on the 3‐hydroxyl group resulted in diminished activity. For example, various saccharide substitutions on the 3‐OH of quercetin, as in quercitrin, isoquercetin, hyperoside, isoquercitroside, and rutin, decreased their activity compared with quercetin. This may be due, at least in part, to the steric factors of the saccharide substituents. In contrast, for flavan‐3‐ols, galloylation on the 3‐OH group increased activity, as evidenced by the slightly improved senolytic activity of EGCG and GCG compared with cyanidin. Unlike flavones or flavonols, the saturated C2‐C3 bond in the flavan‐3‐ols EGCG and GCG disrupts extended conjugation, which may allow greater tolerance of the steric hindrance of the galloyl moiety at the 3‐OH, thus increasing their senolytic activity compared with dihydromyricetin. Additionally, for the flavan‐3‐ol scaffold, stereoisomerism appeared to have little effect on senolytic activity, as the activities of GCG and EGCG are comparable.

### 2.3 SAR‐Guided Iterative Design of Optimized Flavonoid Senolytics

The SAR analysis underscored the importance of the flavone scaffold in developing senolytic FAs, with luteolin emerging as the most potent and selective compound in the preliminary screening. Based on the SAR information, we hypothesized that the absence of a hydroxyl group at the 3‐position in the C ring, combined with the presence of unsubstituted 3′,4′‐dihydroxyl groups in the B ring, could be responsible for the enhanced senolytic activity and selectivity observed in luteolin. We conducted an iterative process of structural optimization, encompassing drug design, chemical synthesis, biological evaluation, and further SAR analysis to identify novel senolytic FAs with improved efficacy and selectivity.

In total, we designed, synthesized, and tested 35 novel flavonoid analogs for senolytic activity in senescent *Ercc1* ^−/−^ MEF cells and for selectivity against non‐senescent WT MEF cells (Figure 1C). The majority of these analogs demonstrated significantly enhanced senolytic activity compared to fisetin or luteolin. These results also highlighted the superiority of the flavone scaffold over the flavonol structure. The SAR analysis provided additional insights consistent with the preliminary findings. For instance, compounds featuring electron‐donating groups on the A ring (compounds 4–7) were more active than those with electron‐withdrawing groups (compound 3). Moreover, substituents at the 6‐position on the A ring were generally more favorable for senolytic activity than those at the 7‐position (compound 7 > compound 6, compound 9 > compound 8). These observations led us to design additional FAs with 6‐substitutions, including aliphatic, alicyclic, and aromatic groups.

Among the acyclic aliphatic substituents, bulky tert‐butyl (compound 17) and isopropyl (compound 18 or SR29384) groups notably enhanced the senolytic activity of the flavonoid analogs. The isopropyl‐substituted compound SR29384 emerged as the most selective senolytic agent among all designed FAs. The introduction of five‐ or six‐membered all‐carbon alicyclic substituents (compounds 22, 24, and 25) further boosted the senolytic activity. Notably, SR31133 (compound 22) exhibited the highest potency, with a senolytic EC_50_ value of 0.8 μM, resulting in a more than 27‐fold improvement in senolytic activity compared to fisetin. However, it had reduced specificity for senescent cells compared to SR29384. Substituted phenyl (compounds 27–33), pyridine (compounds 34 and 35), or benzyl (compound 36) groups at the 6‐position of the A ring were tolerated but exhibited lower selectivity. Furthermore, the presence of free 3′,4′‐dihydroxyl groups in the B ring proved critical for senolytic bioactivity, as substituting these hydroxyl groups (compound 18 or SR29384) with methoxy groups (compound 37) significantly reduced both senolytic activity and selectivity.

Collectively, our phenotype‐guided drug design efforts led to the successful discovery of two promising senolytic FAs: SR29384, the most selective, and SR31133, the most potent. In comparison, fisetin has a diphenylpropane flavonol scaffold with four hydrogen bond donors and a 3‐hydroxy group in the central ring, whereas the two FAs possess a flavone scaffold lacking the 3‐hydroxy group and have only two hydrogen bond donors. As a result of these structural modifications, SR29384 and SR31133 exhibited 2.9‐ and 27.5‐fold greater senolytic activity than fisetin, respectively (Figure 1C).

Regarding the physicochemical and molecular properties, both FAs exhibited increased lipophilicity, with WLogP values of 3.99 for SR29384 and 4.53 for SR31133, compared to 2.28 for fisetin (Figure S2A). The two FAs have lower topological polar surface area (TPSA) values than fisetin, each less than 90 Å^2^ , consistent with physicochemical properties associated with better permeability of the blood–brain barrier (BBB) (van de Waterbeemd et al. 1998). Moreover, both FAs demonstrated better computational molecular properties compared to fisetin in terms of drug score, drug‐likeness, LD_50_ values, and predicted toxicity, including a substantially reduced probability of carcinogenicity (Figure S2A and Data S1). Although these in silico predictions suggest a favorable safety profile, they are preliminary and do not definitively rule out the potential for long‐term carcinogenicity, which requires further empirical longitudinal evaluation.

### 2.4 Broad Spectrum Senolytic Activity of the Optimized Flavonoid Analogs

To further validate the senolytic activity of the newly designed FAs SR29384 and SR31133, we assessed their potential across a range of senescent cell models induced by various inducers. These models include *Ercc1* ^−/−^ MEFs, WT MEF cells subjected to oxidative (20% O_2_, or H_2_O_2_) or genotoxic (etoposide) stress, human IMR90 fibroblast cells induced to senescence by genotoxic stress (etoposide), and human umbilical vein endothelial cells (HUVEC) undergoing replicative senescence. The results showed that both SR29384 and SR31133 effectively reduced the number of C_12_FDG‐positive senescent cells in these senescent cell models (Figure S2B,D). Beyond SA‐β‐gal activity (Figure 1D), the senolytic efficacy of these compounds was further validated by assessing their effects on key senescence and SASP markers by RT‐qPCR. Consistent with the phenotype‐based data, treatment with SR29384 and SR31133 resulted in significant downregulation of *Cdkn2a* (*p16* ^*Ink4a*^), *Cdkn1a* (*p21* ^*Cip1*^), and *Il6* compared to fisetin in senescent MEFs (Figure S2C). Therefore, we selected SR29384, the most selective, and SR31133, the most potent, for further preclinical evaluations in mice.

### 2.5 Mouse Studies Demonstrate Reduction of Tissue Senescence and Improvement in Healthspan‐Associated Markers

To assess the in vivo senolytic potential of SR29384 and SR31133, we used acute treatment of old WT C57BL/6J mice (28 months) in which fisetin would not be expected to be significantly effective according to previous studies (Yousefzadeh et al. 2018). Instead of the 100 mg/kg dose used in earlier fisetin studies, the old wild‐type C57BL/6J mice were acutely treated with a lower dose of 20 mg/kg body weight of the compounds for five consecutive days. This lower dose was selected as a stringent comparison to determine whether the optimized analogs could show improved activity under conditions where fisetin would be expected to have limited efficacy. Mice were euthanized 2 days after the last administration and tissues were collected for analysis (Figure 2A). The RT‐qPCR analysis of different tissues found that fisetin had a marginal effect on senescence and SASP. However, the two FAs, SR29384 in particular, significantly decreased the expression of senescence markers *p16* ^*Ink4a*^ and *p21* ^*Cip1*^ as well as multiple SASP factors, such as *Il6*, *Il1β*, *Mcp1*, and *Pai1*, in different tissues including kidney, brain, lung, spleen, fat, and muscle (Figure 2B,C, Figures S3 and S4). Overall, these data support tissue‐ and marker‐dependent senolytic activity, with SR29384 outperforming fisetin for some endpoints, but not showing uniform superiority across all tissues.

**FIGURE 2:**
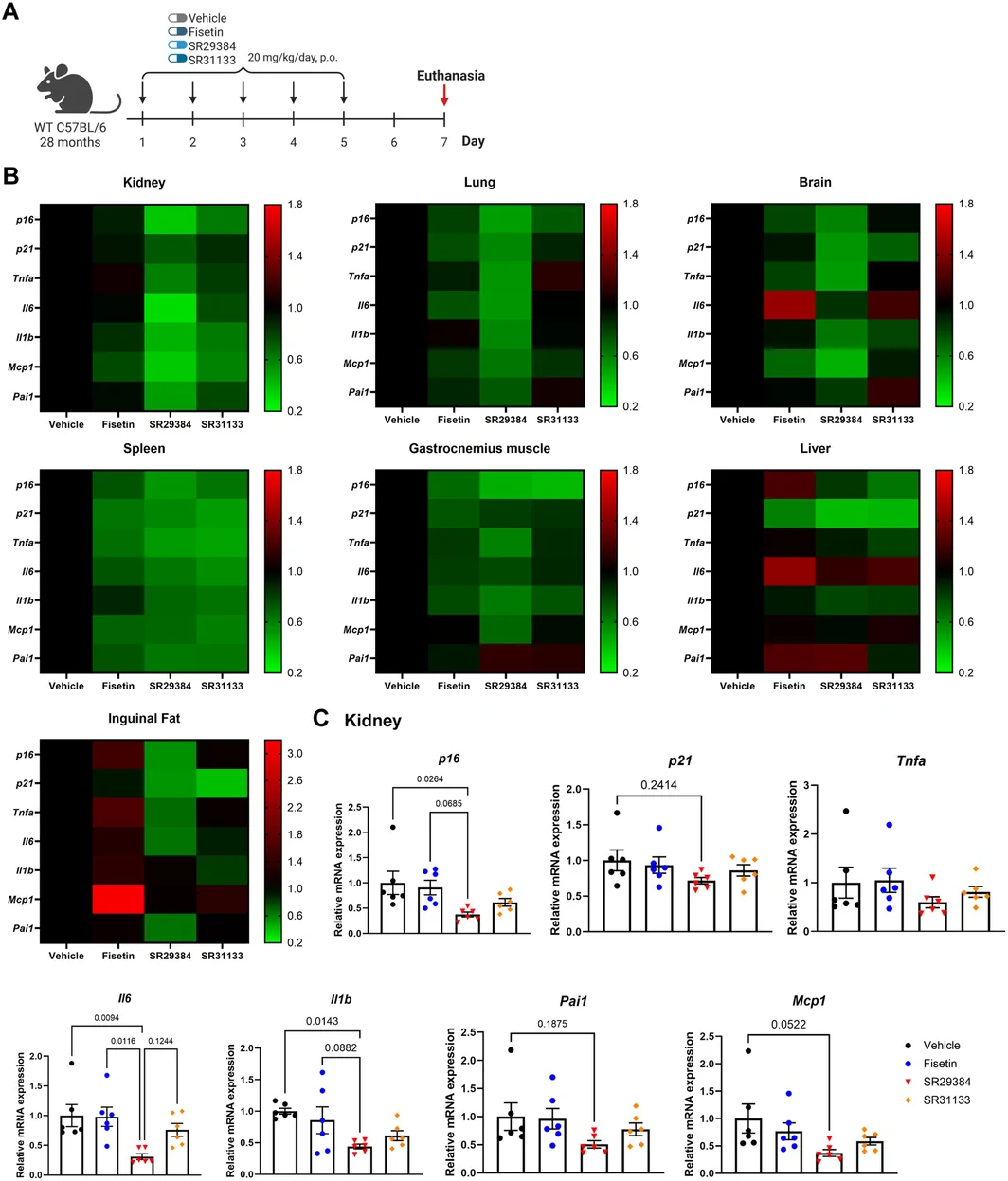
Acute treatment with the flavonoid analogs reduced tissue senescence in naturally aged mice. (A) At 28 months of age, wild‐type C57BL/6J mice were administered vehicle, fisetin, SR29384, or SR31133 for five consecutive days by oral gavage at a dosage of 20 mg/kg/day. Mice were euthanized 2 days after the final dose, and tissues were collected for analysis. (B) Heatmap showing mRNA expression changes of senescence and SASP factors in various tissues following treatment with vehicle, fisetin, SR29384, or SR31133. (C) RT‐qPCR analysis of kidney tissue showing relative mRNA expression of senescence and SASP markers across treatment groups. Error bars represent the standard error of the mean (SEM); *n* = 6 mice per group.

The most effective fisetin analog in the acute mouse study, SR29384, was selected for testing in *Ercc1* ^−/Δ^ mice to determine its effect on tissue senescence and healthspan. *Ercc1* ^−/Δ^ mice were treated with SR29384 at 20 mg/kg by oral gavage three times per week for 5 weeks (Figure 3A). Health assessments were conducted weekly to score age‐related symptoms, including tremor, kyphosis, dystonia, ataxia, gait disorder, and body condition. Results showed that SR29384 effectively suppressed the composite score of aging symptoms, particularly dystonia and ataxia (Figure 3B). There were no significant differences in the composite aging score at week 16. Gene expression analysis in tissues showed a significant reduction or trend towards reduction in senescence and SASP factors across multiple tissues, including the kidney, liver, lung, and brain (Figure 3C). Although direct pharmacokinetic measurements were not performed in this study, the systemic reduction of senescence markers in diverse organs, including the brain, serves as functional confirmation that SR29384 possesses sufficient oral bioavailability to engage its targets in vivo. Collectively, these in vivo studies support the therapeutic potential of SR29384 as a novel and potent senolytic agent, capable of reducing senescence markers, and alleviating selected healthspan‐associated metrics, particularly dystonia and ataxia in mice.

**FIGURE 3:**
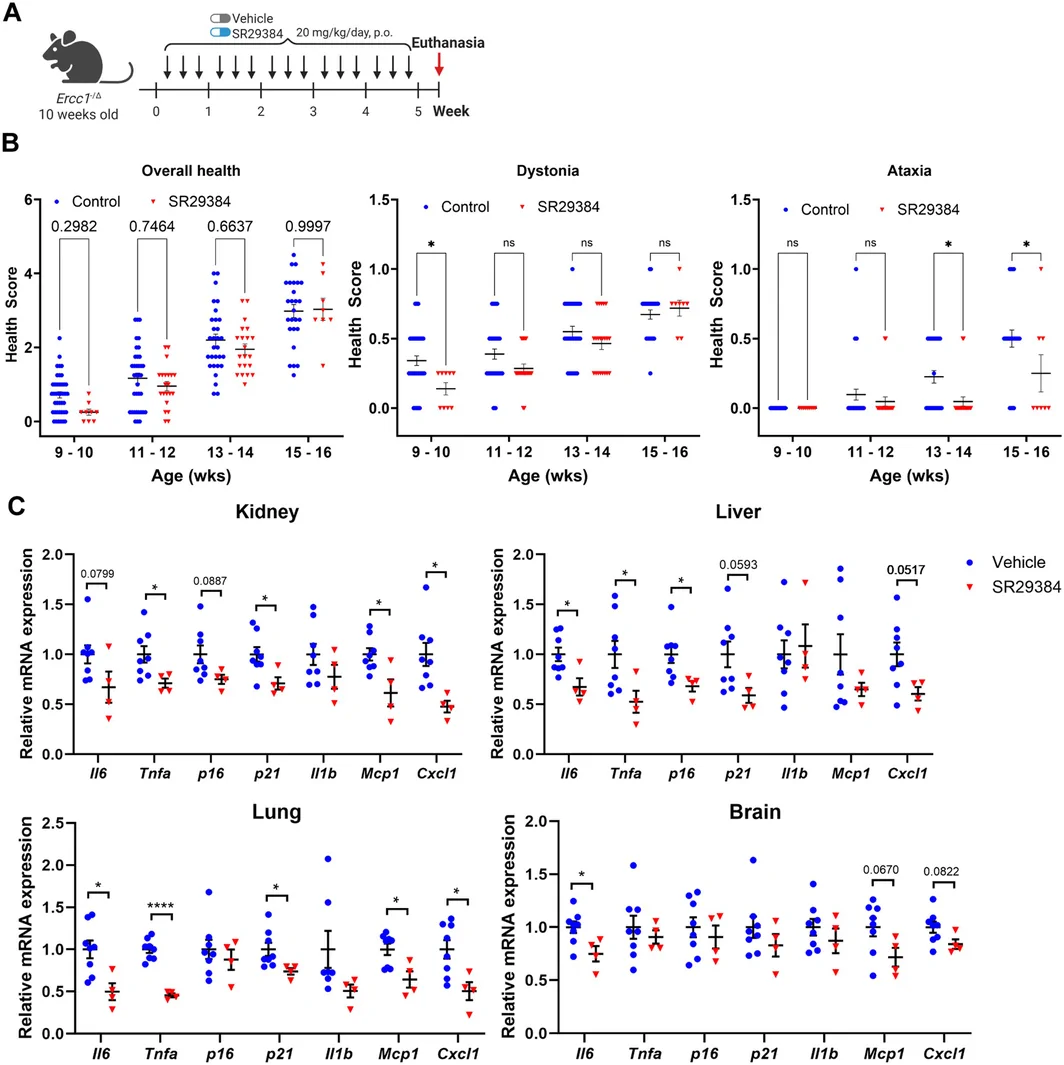
SR29384 improved selected healthspan‐associated metrics and reduced tissue senescence in the *Ercc1* ^−/Δ^ progeroid mouse model. (A) At 10 weeks of age, *Ercc1* ^−/Δ^ progeroid mice were treated with SR29384 or vehicle by oral gavage three times per week for 5 weeks. Mice were euthanized 2 days after the final dose, and tissues were collected for further analysis. (B) Health assessments were performed weekly to score diverse aging‐associated symptoms including tremor, kyphosis, dystonia, ataxia, gait disorder, and body condition. (C) RT‐qPCR analysis of senescence and SASP markers in tissues collected from vehicle‐treated and SR29384‐treated mice. Error bars represent SEM; *n* = 8 vehicle‐treated mice and *n* = 4 SR29384‐treated mice.

### 2.6 Integrative Transcriptomic, Computational, and Biochemical Analyses Implicate PARP1, BCL‐xL, and CDK2 as Potential Targets

To elucidate the mechanisms underlying the enhanced senolytic activity of FAs SR29384 and SR31133, we employed a multidisciplinary approach. First, we examined whether differences in antioxidant capability could be a contributing factor. Using the DPPH radical scavenging assay (Brand‐Williams et al. 1995), we compared the antioxidant activity of fisetin and the two analogs. The results showed that all compounds exhibited similar free radical scavenging activity, as demonstrated by their comparable IC_50_ values (Figure S2E). This finding suggests that the enhanced senolytic activity of SR29384 and SR31133 is not due to differences in antioxidant properties, prompting further investigation into their molecular targets.

Next, we conducted bulk RNA‐Seq analysis to identify gene expression changes and key signaling pathways in non‐senescent (NS) and etoposide‐induced senescent (S‐Eto) cells following treatment with fisetin, SR29384, and SR31133 (Figure S5A,B). First, we confirmed enrichment of the SenMayo gene set (Saul et al. 2022) in the S‐Eto cells (NES: 1.755). Then, we observed a reduction of SenMayo enrichment in the S‐Eto cells treated with SR29384 (NES: −1.597) and SR31133 (NES: −1.807); however, no significant change in enrichment was observed with fisetin at the dosage tested (Figure S5C and Data S2). To better understand the underlying biological processes, we carried out Gene Set Enrichment Analysis (GSEA) using the Hallmarks database to identify pathways associated with the differentially expressed genes (DEGs). The top pathways activated by SR29384 and SR31133 were associated with hypoxia, p53 signaling, and multiple stress signaling pathways, while downregulated processes included G_2_/M transition, E2F targets, and DNA replication processes (Figure S5D and Data S2). Venn diagrams and volcano plots were used to display the DEG overlap and treatment‐specific transcriptomic changes that supported these enrichment analyses (Figure S5A,B).

To further explore the possible molecular mechanisms, we used a sequence of in silico structure‐based machine learning models and network analyses. First, we used PathwayMap, a deep self‐normalizing convolutional neural network (DSNNN) machine learning model, to predict compound‐pathway interactions (Jimenez et al. 2019). In addition to the two FAs, we included 7 reported senolytics for comparison, including fisetin and luteolin. PathwayMap probability scores for interactions with human biological pathways reveal that SR29384 and SR31133 had highly similar interaction signatures (Figure S6A–C). In contrast to fisetin, which clustered closely with quercetin, SR29384 and SR31133 did not have a strong interaction probability for human longevity pathways. Instead, SR29384 and SR31133 cluster with luteolin and dasatinib, which were predicted to strongly interact with pathways associated with G_2_/M transitioning, oxidative stress, cellular senescence, ER stress, TP53 signaling, ESR signaling, and caspase activation. Additional data for pathway enrichment using the PathwayMap algorithm are provided in Data S1.

These preliminary interaction probabilities prompted the identification of specific molecular drug targets. We used structural features of the drugs to predict such drug‐protein interactions in the human druggable proteome using SwissTargetPrediction, a similarity‐based algorithm trained with curated experimental ligand‐receptor binding assays (Gfeller et al. 2014). To reduce false positive targets, we included 10 compounds that showed no senolytic activity as negative controls (“NC” in Data S1). This NC filtering excluded targets shared with non‐senolytic FAs and reduced biases introduced by the scaffold. After filtering, SR29384 and SR31133 were found to target 49 and 44 proteins, respectively, with 29 overlapping targets (Figure 4A). To understand the biological functions of these targets, we constructed clustered protein–protein interaction (PPI) networks of the shared and exclusive targets for the two candidates. Targets shared between SR29384 and SR31133 grouped into three major clusters: p53 signaling and cell cycle regulation, estrogen receptor (ESR) activity, and PARP signaling. In addition to these shared targets, nodes exclusive to SR29384 were associated with inflammatory signaling and ECM remodeling. Conversely, SR31133‐exclusive targets were enriched for histone modification processes, particularly HDAC family proteins (Figure 4B). These enrichment trends for the networks corroborated our previous findings from the DSNNN‐based PathwayMap analysis. The shared target network was enriched for pathways associated with G_2_/M transitioning, ESR signaling, stress response, and apoptosis regulation. The SR29384‐exclusive target network was enriched for immune cell activation, ECM disassembly, and response to hypoxia. The SR31133‐exclusive target network was primarily enriched for deacetylation and transcriptional regulation (Figure S6D). Interestingly, after NC‐filtering, SR29384 and SR31133 shared more targets with each other than with their parent compounds fisetin or luteolin (Figures S6A–C and S7A). Similar clustering and enrichment approaches also distinguished the two candidate FAs from luteolin and fisetin by exclusive targets enriched in processes associated with inflammatory signaling, unsaturated fatty acid metabolism, and apoptosis (Figures S7B and S8A,B). Further details on SwissTargetPrediction scores are available in Data S3.

**FIGURE 4:**
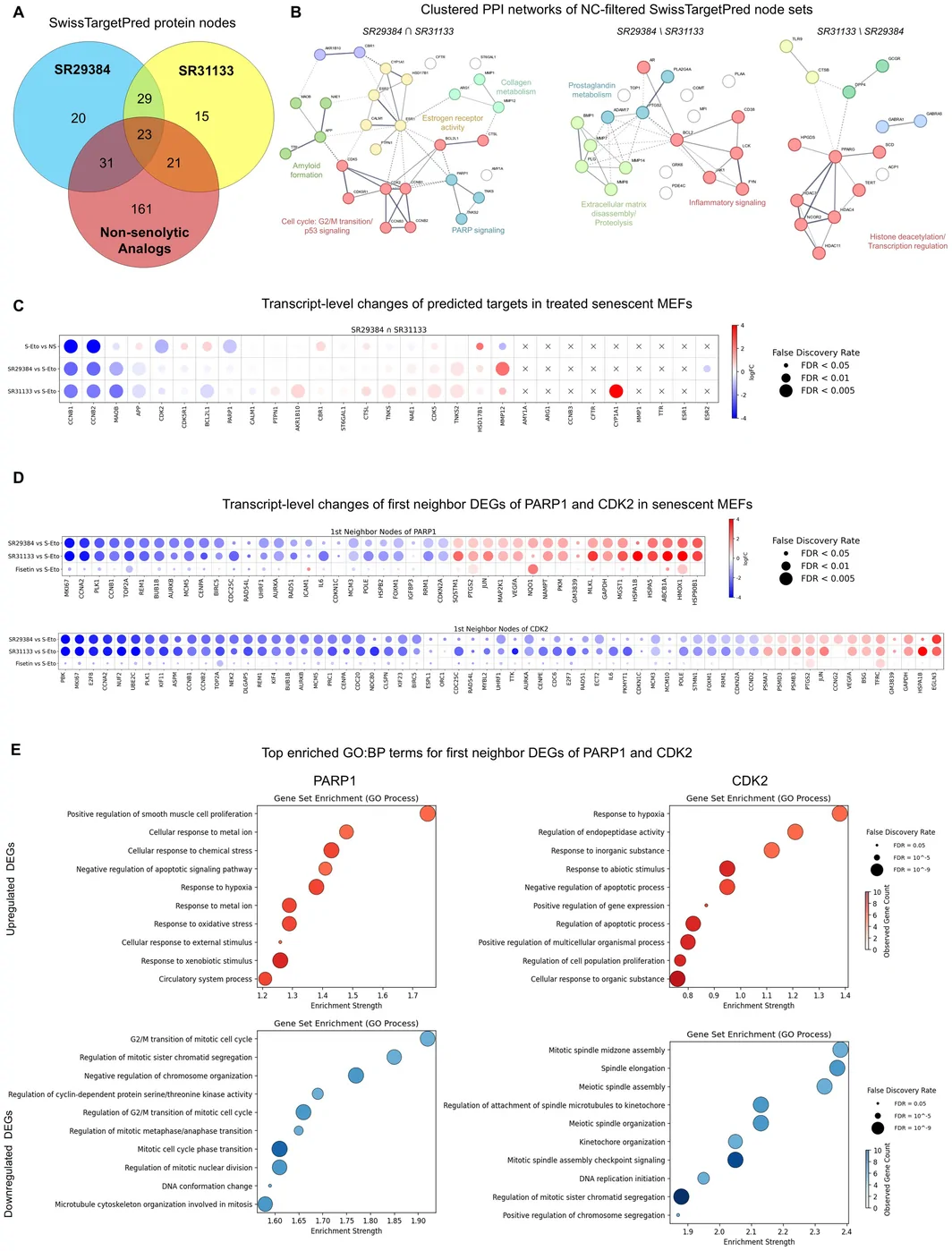
SR29384 and SR31133 share targets in the PARP1 and CDK2 protein interactomes. (A) Venn diagram depicting overlaps between targets for SR29384, SR31133, and unique targets filtered for negative control FAs (NC) using SwissTargetPrediction scores. (B) k‐means clustered STRING PPI maps constructed from shared and exclusive targets for SR29384 and SR31133. Target sets were filtered to exclude targets shared with NCs. Nodes are clustered based on degree centrality. The top enriched GO‐terms for each cluster are used as labels. Connecting edges are defined as level of interaction confidence (probability > 0.9). (C) Bubble plot grid depicting changes in mRNA expression of predicted shared targets in etoposide‐induced senescence in MEFs, and senescent MEFs treated with SR29384 and SR31133. (D) Bubble plot grid depicting changes in mRNA expression of 1st neighbor node DEGs of *PARP1* and *CDK2* in senescent MEFs treated with SR29384, SR31133, and fisetin. (C, D) Genes are sorted by log_2_FC values in the SR29384 versus S‐Eto dataset. Color and sizes of bubbles correspond to a set scale for log2FC and FDR values; “x” symbols represent genes not detected in dataset (CPM ≤ 0.5). (E) Ranked bubble plot depicting top 10 enriched pathways (GO Biological Processes) for upregulated and downregulated (|log_2_FC| > 1, FDR < 0.05) 1st neighbor DEGs for *PARP1* and *CDK2* in senescent MEFs treated with SR29384 and SR31133. Bubble color intensity and sizes correspond to the number of observed genes in GO term and FDR values (< 0.05).

Although these sets of targets provided us with an initial array of putative targets, they required experimental support. For this, we first used detectable mRNA levels (CPM > 0.5) of each target in our RNA‐Seq experiments as a proxy for cellular expression of targets. Nodes such as *ESR1*, *ESR2*, and *TTR* that did not meet this criterion were excluded (Figure 4C). Because small molecules often alter protein activity without changing transcription of the target gene itself, we reasoned that direct protein targets may not show major mRNA changes after short‐term FA treatment. However, for selective targeting of senescent cells, we expected that genes involved in senescence‐related pathways would be altered during the senescence program. Using this rationale, we filtered for targets with |log_2_FC| > 1 and FDR < 0.05 in the non‐senescent (NS) versus etoposide‐induced senescent (S‐Eto) MEF RNA‐Seq datasets, with no significant change in expression between SR29384 or SR31133 treatment and the S‐Eto groups (Figure 4C, Data S4). This analysis identified *PARP1*, *CDK2*, *CDK5R1*, *HSD17B1*, and BCL‐xL (transcript name *BCL2L1*) as potential targets shared by the candidate FAs. Similarly, exclusive targets exhibiting this signature were *TOP1*, *COMT*, and *GRK6* for SR29384, and *TERT*, *HDAC4*, and *HDAC11* for SR31133 (Figure S9A, Data S4).

If these shortlisted proteins are direct targets of the candidate FAs, they should have a substantial impact in perturbing their local gene network neighborhoods. For this, we isolated subnetworks consisting of nodes from 1st neighbor interactor genes for each of the query proteins from a parent network of all DEGs from the FA‐treated RNA‐Seq datasets. Based on the number of DEGs in the local neighborhood and perturbation signatures, we determined PARP1, CDK2, and BCL‐xL as the best potential protein targets shared by the two FAs (Figure 4D and Figure S9B). Indeed, the expression‐level changes of DEGs in their 1st shell neighborhoods showed significant alterations without any changes in the targets themselves at the transcript level (Figure 4D and Figure S9C). Interestingly, there was minimal change detected for these genes in fisetin‐treated senescent MEFs. Conversely, there was a strong resemblance in magnitude and directionality of differential expression of the DEGs between SR29384 and SR31133 treatments. Similar first neighbor interaction maps for the predicted SR29384‐ and SR31133‐exclusive targets were constructed (Figure S9D). Furthermore, perturbations in shared FA target neighborhood DEGs functionally enriched many processes that mirror ontologies from both our RNA‐Seq analyses and structure‐based predictions (Figures S5D and S6D). This indicates that changes in first neighbor DEGs of PARP1, BCL‐xL, and CDK2 alone are sufficient to mimic the overall transcriptomic enrichments in senescent cells treated with the two FAs. These processes include DNA damage response and repair, G_2_/M cell cycle transitioning, response to hypoxia and oxidative stress, and anti‐apoptotic processes (Figure 4E).

Additionally, individual queries of neighbor nodes using StringDB suggest the directional signatures of the DEGs to be associated with inhibited antagonists and upregulated agonists/co‐expressors of PARP1, BCL‐xL, and CDK2 proteins. This suggests that SR29384 and SR31133 may act as inhibitors of these target proteins. To support this, we performed molecular docking simulations for the three top target proteins to assess the energetic and conformational feasibility of the candidate FAs to function as inhibitor compounds. Each set of ligands docked into pre‐defined inhibitory conformational spaces includes a collection of random decoy molecules (DUDs) as technical negative controls, a native substrate, two FAs with no experimental senolytic activity as experimental negative controls, two natural senolytic FAs (fisetin and luteolin), and three standard inhibitors of each protein. A list of crystal receptor structures, native substrates, positive controls, and relevant references is included in Data S5.

In the case of the PARP1 catalytic cavity, an ideal competitive inhibitor should bind with a stronger binding affinity than the activating co‐factor NAD+. The best docked ligand conformations for SR31133, SR29384, and luteolin, but not fisetin, have stronger interactions than native substrates or non‐senolytic FAs within the receptor cavity (Figure 5A,C). This was further elucidated by the ligand orientations within the binding cavity, and the interaction characteristics of ligand atoms with surrounding amino acid residues. Fisetin, luteolin, SR29384, SR31133, and the top‐scoring standard talazoparib shared key favorable hydrogen bonding events with residues including GLY863, GLY904, GLN759, TYR889, TYR896, and SER904. Additionally, a set of hydrophobic interactions with TYR889, TYR896, ALA898, and TYR907 contributed to the favorable binding profiles. However, two donor‐donor clashes with HIS862 and MET890 residues inhibit fisetin from strongly binding to the cavity. In contrast, the benzamide core of talazoparib established a strong halogen bond with GLU988, a feature absent in the flavonoid analogs (Figure 5B).

**FIGURE 5:**
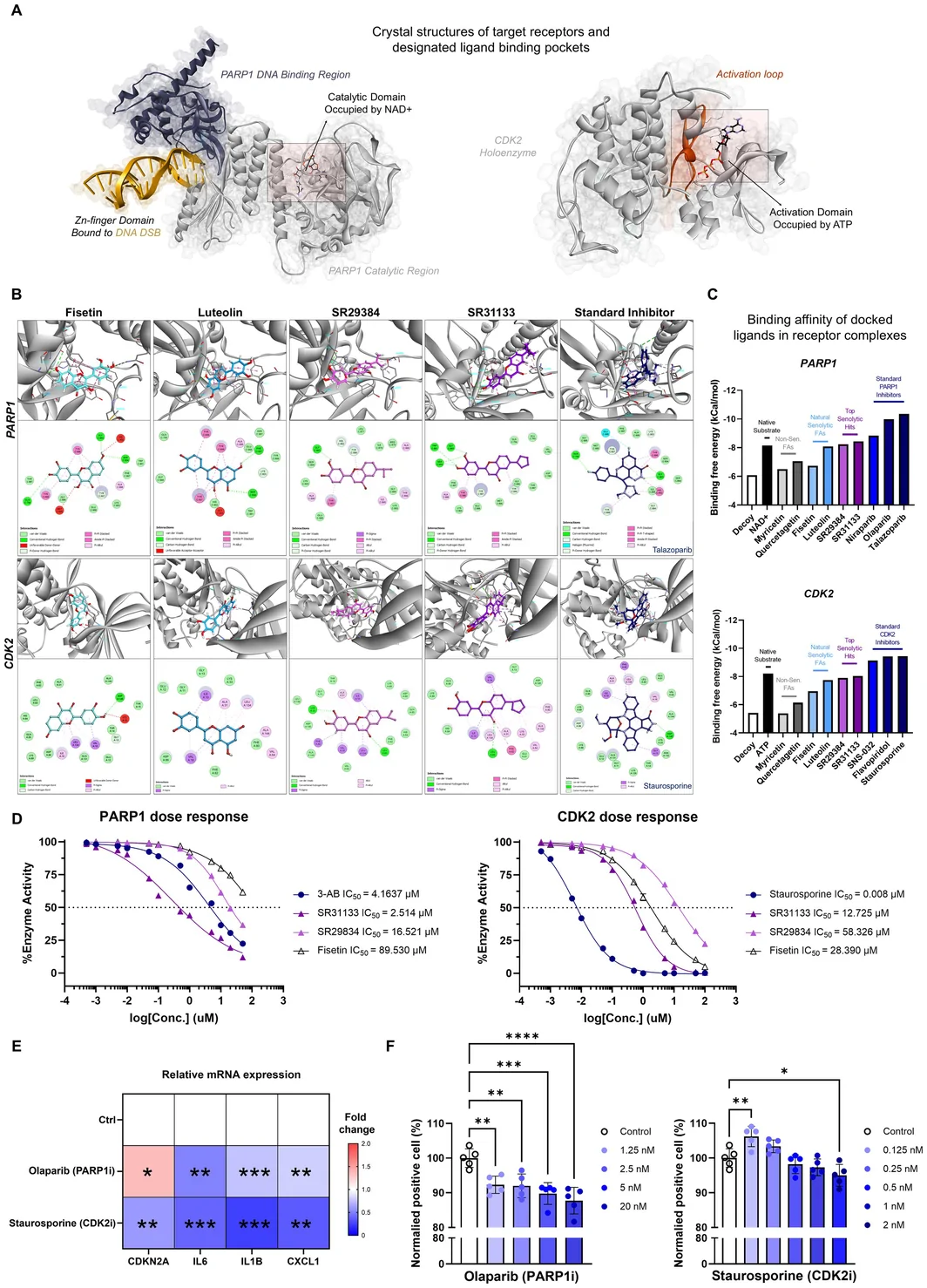
SR29384 and SR31133 are direct inhibitors of CDK2 and PARP1. (A) 3D molecular model depictions of the receptor protein crystal structures used for docking simulations with surrounding native templates used for search area definition. (B) Flexible molecular docking results of predicted target proteins with candidate FAs and control compounds. Native substrate or ligand and known inhibitor or activator drug for each protein target were used along with a randomly generated structural decoy/DUD molecule. 3D and 2D interaction plots of ligands in the binding pockets with interacting pocket amino acid residues. The best‐scoring standard inhibitor for each target was represented; ligand‐residue interaction legends are provided under the 2D plots. (C) Binding free energy scores of best‐fitting conformers for each ligand within the selected receptor binding pockets; lower binding free energies correspond to stronger ligand‐receptor interactions. Color identifiers correspond to panel (B). (D) Enzyme inhibition assays validating predicted target interactions. Recombinant PARP1 and CDK2/Cyclin A2 were incubated with increasing concentrations of SR29384 and SR31133, and residual enzymatic activity was quantified using colorimetric (PARP1) and luminescence‐based (CDK2) assays respectively. Dose–response curves were generated from normalized % activity values and fitted by nonlinear regression to determine compound potency along with established reference inhibitors and fisetin. (E) PARP1 or CDK2 inhibition suppresses senescence‐associated genes in senescent IMR90 cells treated with a PARP1 inhibitor or CDK2 inhibitor for 48 h. (F) Quantification of C_12_FDG‐positive cells in senescent IMR90 cells treated with PARP1 inhibitor or CDK2 inhibitor for 48 h. The SA‐β‐gal staining data are presented as the percentage of C_12_FDG‐positive cells among total Hoechst‐positive nuclei. The PARP1 inhibitor significantly decreased C_12_FDG positivity across multiple concentrations, whereas CDK2 inhibitor showed no apparent reduction compared with control. Data are normalized to the control group.

In the CDK2 activation domain, competitive inhibition of ATP‐binding will disrupt the cyclin binding and phosphorylation in the activation loop. Both SR29384 and SR31133 were predicted to outcompete the native substrate and bind to the ATP‐binding cavity. The strongest inhibitor for CDK2 is staurosporine, which relies on an array of weak hydrophobic interactions with ILE10, GLY13, VAL18, ALA31, PHE82, and LEU134. Luteolin, SR29384, and SR31133 shared many of these hydrophobic interactions, even establishing more interactions with LYS33, VAL64, PHE80, and ALA144. The best docked pose of fisetin had the lowest number of hydrophobic interactions, with unfavorable donor‐donor clashes again making its ability to inhibit CDK2 energetically unfavorable.

When docked into the navitoclax/BH3 binding site of BCL‐xL, the native BAK peptide had a strong binding affinity threshold that is overcome by the standard inhibitors, but not fisetin or the FAs. The binding affinity for the standard A‐1331852 was substantially enhanced by an established cationic bond with ARG100, which was absent in the flavonoid ligands relying on hydrophobic interactions (Figure S10A–C).

Taken together, our integrated approach combining in vitro antioxidant activity assays, transcriptomics, machine learning algorithms, in silico structure‐based predictions, and network perturbation analyses of gene expression in senescent cells identified PARP1, CDK2, and possibly BCL‐xL as key molecular targets underlying the senolytic activities of SR29384 and SR31133. To determine if indeed the FAs targeted these identified proteins, the ability of the FAs to inhibit their activities was examined. In accordance with our predictions, both analogs strongly inhibited PARP1 NAD+‐dependent ribosylation activity more effectively than fisetin, with SR31133 (IC_50_ = 2.51 μM) even outcompeting the standard PARP1 inhibitor 3‐AB (Figure 5D). Similarly, CDK2 kinase activity and substrate peptide phosphorylation were inhibited by SR31133 (IC_50_ = 12.73 μM) and less strongly by SR29384 (IC_50_ = 58.33 μM) (Figure 5D), supporting PARP1 and CDK2 as potential shared mechanistic targets. However, neither FA nor fisetin altered BCL‐xL binding to its target ligand substrate at 100 μM concentrations (Figure S10D). These results suggest that the observed alterations in the BCL‐xL interaction neighborhood following FA treatment are at least unlikely to result from direct binding or catalytic inhibition of BCL‐xL.

Finally, we tested whether pharmacologic perturbation of PARP1 or CDK2 could modulate senescence‐associated phenotypes. In senescent IMR90 cells, the PARP1 inhibitor olaparib significantly reduced expression of multiple SASP genes and modestly reduced the percentage of SA‐β‐gal‐positive cells (Figure 5E,F). Similarly, the CDK2 inhibitor staurosporine reduced expression of SASP genes as well as *p16* ^*Ink4a*^, although it did not reduce the percentage of SA‐β‐gal‐positive cells under these conditions (Figure 5E,F). These data support that both CDK2 and PARP1 may have functional roles in senescent cell survival and SASP expression, and suggest that the senolytic activity of FAs may be mediated through targeting both CDK2 and PARP1 and possibly other pathways.

## 3 Discussion

The discovery of senolytic drugs remains challenging due to the limited identification of specific molecular targets, with BCL‐2/BCL‐xL being one of the few identified. In this study, we aimed to develop novel flavonoid‐based senolytics using fisetin as a starting point, despite its largely unknown molecular targets. To address this challenge, we employed a phenotypic drug discovery approach, which focuses on identifying compounds based on their functional effects rather than predefined target interactions (Berg 2021; Moffat et al. 2017; Vincent et al. 2022). This strategy allowed us to obtain valuable structure–activity relationship (SAR) insights, which guided the subsequent drug design process. As a result, we successfully identified two novel flavonoid analogs, SR29384 and SR31133, with significantly enhanced senolytic activities compared to fisetin. These compounds exhibit broad‐spectrum activity across diverse senescent cell types, highlighting their potential to treat a wide array of senescence‐associated diseases, including aging‐related pathologies, fibrosis, and certain cancers where senescence plays a critical role. The potential translational relevance of SR29384 and SR31133 was demonstrated in preclinical models, where they effectively reduced tissue senescence at relatively low doses and improved selected healthspan‐associated parameters in accelerated aging mice. These findings establish SR29384 and SR31133 as promising candidates for the development of safe and effective senotherapeutics.

During our initial phenotypic drug screening, we evaluated the senolytic activities of various flavonoids and identified luteolin as a more potent natural senolytic than fisetin. Consistent with this, previous studies have shown that luteolin can suppress H_2_O_2_‐induced senescence in HEI‐OC1 cells (Zhu et al. 2021) and TNF‐α‐induced senescence in human nucleus pulposus cells (Xie et al. 2022). Additionally, luteolin is the primary flavonoid in Haenkenium extract, which has been reported to reduce senescence induced by aging or chemotherapy (Zumerle et al. 2024). Our screening also found that neither fisetin nor quercetin exhibited significant senolytic activity at lower concentrations (Figure 1A). Specifically, fisetin showed only weak senolytic activity, at least at lower concentrations, in senescent *Ercc1* ^−/−^ MEFs and minimal senolytic effects on other tested cell types, including senescent IMR90 cells, WT MEFs, and HUVECs. Some studies have reported the potential of fisetin as a senolytic agent. For example, we reported that fisetin reduced the burden of senescent cells and extended lifespan in naturally aged and accelerated aging mouse models (Yousefzadeh et al. 2018). However, fisetin, like other known senolytics, is not a pan‐senolytic, displaying varying senolytic activity across different cell types and experimental conditions. For example, fisetin was found to kill senescent MEFs, but not senescent human melanoma SK‐MEL‐103 cells (Annunziata et al. 2023). Additionally, fisetin, along with quercetin, was found to minimally reduce *p16* ^*Ink4a*^‐positive fibroblasts isolated from fibrotic mouse lungs (Lee et al. 2024), suggesting the senolytic activity of fisetin is context‐dependent in vivo. It is also important to note that fisetin and other flavonoids have a wide range of cellular and systemic biological effects, including anti‐inflammatory, antioxidant, anticancer, antidiabetic, and neuroprotective activities (Proshkina et al. 2024; Rauf et al. 2023; Ravula et al. 2021; Sundarraj et al. 2018), which may contribute to their gerotherapeutic benefits independent of their specific and direct senolytic properties.

By integrating SAR analysis, transcriptomics, machine learning, network pharmacology, and molecular docking, we not only identified potent senolytics but also uncovered key molecular mechanisms underpinning their activity. Among these, PARP1 and CDK2 were the most pronounced target proteins in our model. PARP1 is widely known for its role in cancer cell survival and DNA damage response processes. PARP inhibitors have been shown to induce a transient “senescence‐like” phenotype in cancer cells (Fleury et al. 2019; Kolacz and Robaszkiewicz 2024), which makes them vulnerable to secondary modes of clearance, such as BCL‐2 and BCL‐xL inhibitors (Fleury et al. 2019; Softah et al. 2023). Paradoxically, non‐tumor senescent cells consistently have dampened expression and activity of PARP1 at the transcript and protein levels (Nehme et al. 2023). Accordingly, MEFs treated with DNA damage agents in our experiment exhibited reduced expression of PARP1 and downstream targets. Suppression of PARP1 with inhibitors has been suggested to reduce inflammatory signaling and induce apoptosis by bypassing PARP‐mediated DNA SSB repair and trapping PARP on cytotoxic DNA complexes (H. Zhu et al. 2020). Both SR29384 and SR31133 directly inhibit PARP1 and significantly blunt neighboring nodes and pathways. This potentially exploits a therapeutic vulnerability in senescent cells by disrupting detection of DNA damage and promoting apoptosis. Similar to PARP1, evidence related to crosstalk between CDK2, senescence, and apoptosis components is conflicting. Although cellular protein inhibitors of CDK2 such as p21^Cip1^ and p27^Kip1^ are associated with inducing senescence in cancer cells, small‐molecule inhibitors of CDK1/CDK2 such as flavopiridol and staurosporine show strong caspase cleavage and apoptosis induction via regulation of E2F and BCL‐xL/MCL‐1 (Jiang et al. 2003; Samuelsson et al. 2002). Importantly, our results show that targeting PARP1 or CDK2 alone with inhibitors is sufficient to significantly reduce senescence signatures.

Another key signature of senescent cells is resistance to apoptosis through upregulation of BCL‐2 family proteins. BCL‐2 and BCL‐xL also have been suggested to directly participate in cell cycle arrest of senescent cells (Martin et al. 2023). Indeed, etoposide‐treated cells in our study showed significant upregulation of BCL‐xL. From our model and network analysis, SR29384 and SR31133 are predicted to alter its neighboring nodes and their functional processes, including regulation of apoptotic processes. However, our in vitro analysis did not reveal direct inhibition of BCL‐xL by the FAs. Nonetheless, this doesn't necessarily prove that the FAs are not more effective in inhibiting BCL‐xL and anti‐apoptotic processes in vivo. Based on transcriptomic signatures, network modeling and the intricate cross‐talk among PARP1, CDK2, and the BCL‐2 family proteins, we speculate that the FAs may contribute to apoptotic clearance and BCL‐xL functionality without direct binding.

Indeed, there is also substantial crosstalk among different targets. For instance, PARP1 is directly phosphorylated by CDK2. PARP1 inhibition or caspase‐mediated cleavage has also been suggested to direct DNA‐damaged cells towards apoptotic processes rather than necrotic cell death by disrupting PARP1‐BCL‐2 and PARP1‐BCL‐xL interactions (Dutta et al. 2012; Yu et al. 2024). BCL‐xL also acts in tandem with CDK2 to inhibit apoptosis, while inhibitors for both synergize and induce apoptotic cell death (Rello‐Varona et al. 2019). A key process consistently enriched throughout the study was the hypoxia response, which was upregulated in senescent cells and further enhanced in MEFs treated with the candidate FAs. Both PARP1 and CDK2 have been reported to directly interact with and regulate HIF1A and hypoxia response in different cell types (Adachi et al. 2001; Zhu et al. 2023). Conversely, hypoxic stress has also been shown to inhibit cell cycle progression and regulate BCL‐xL (Chen et al. 2009; Gardner et al. 2001). Although tissue‐wide hypoxia promotes cellular senescence and survival, cellular increase in hypoxic response is strongly associated with hypoxic death due to mitochondrial dysfunction, ROS generation, and loss of energy‐demanding processes such as protein folding (Chen et al. 2009; Fuhrmann and Brune 2017; Yan et al. 2021). In our transcriptomic and prediction models, these processes are consistently enriched in senescent cells treated with the candidate FAs. Taken together, these results suggest a multi‐hit potential of SR29384 and SR31133 for a robust induction of apoptotic processes in senescent cells encompassing DNA damage response, pro‐death stress signaling, and inhibition of anti‐apoptotic mechanisms (Figure 6).

**FIGURE 6:**
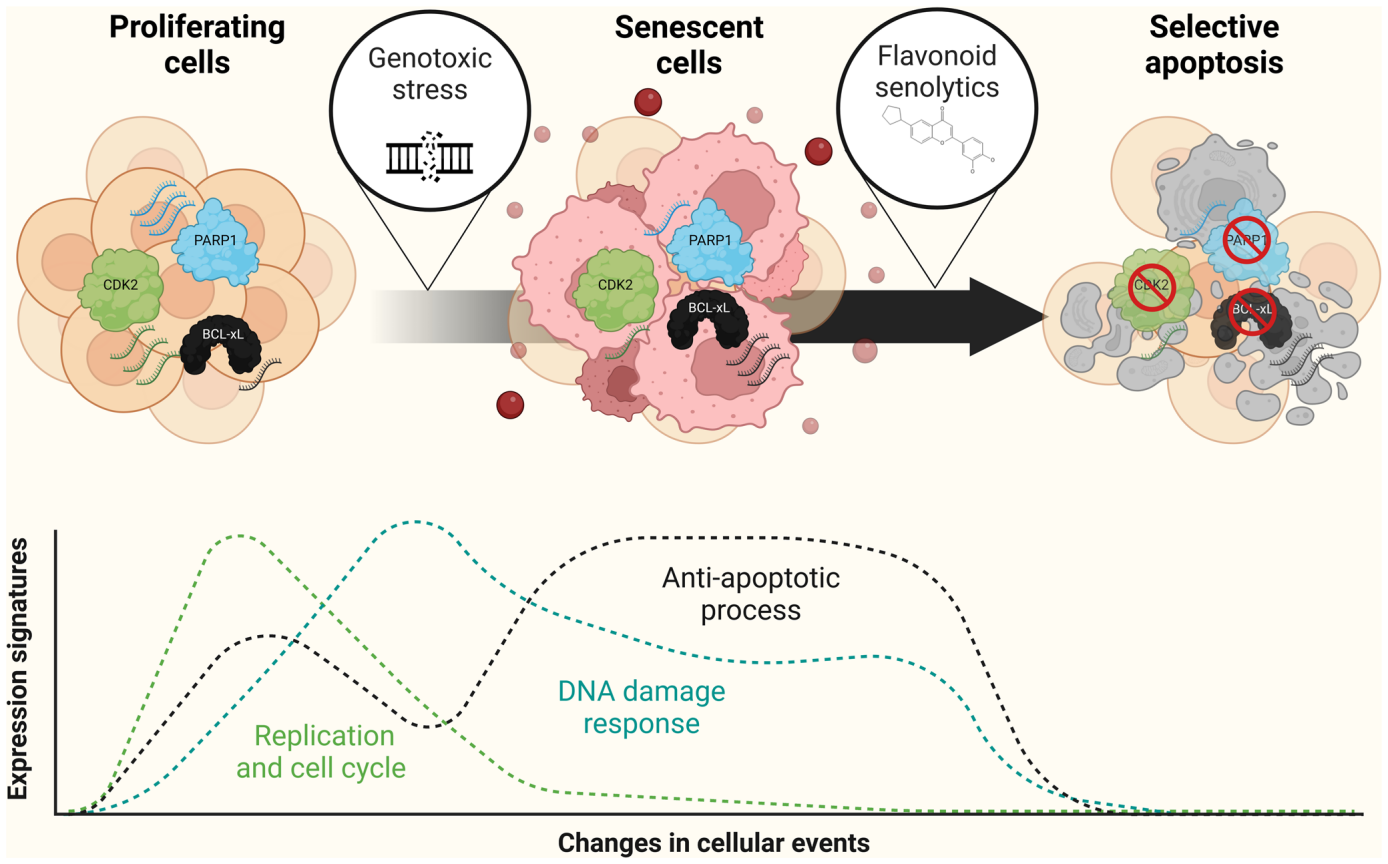
Proposed mechanism of flavonoid senolytic‐mediated apoptotic clearance of senescent cells. Senescent cells, induced by genotoxic stress, exhibit elevated activity of key proteins involved in cell cycle arrest, DNA damage response, and anti‐apoptotic pathways, such as PARP1, CDK2, and BCL‐xL. Flavonoid senolytics (SR29384 and SR31133) selectively target these proteins in senescent cells, disrupting their pro‐survival processes and promoting apoptosis, while sparing proliferating cells. The lower panel shows changes in cellular expression signatures, where replication and cell cycle processes decline, DNA damage response escalates, and anti‐apoptotic signaling is elevated in senescent cells, which is later suppressed by the senolytic action of flavonoid compounds, leading to cell death.

Interestingly, in addition to shared targets for cell cycle regulation and anti‐apoptosis, targets exclusive to SR29384 were enriched for pathways involving prostaglandin and other pro‐inflammatory mediators, ECM disassembly, and activation signaling for many immune cells. All of these processes are routinely associated with the release of the SASP and pro‐inflammatory signaling, characteristic of senescent cells and their microenvironment (Marin et al. 2023; Mavrogonatou et al. 2019). This might present important clues to the remarkable selectivity of SR29384 observed in our senolysis experiments. Conversely, SR31133‐exclusive targets were strongly enriched for histone deacetylation processes. HDAC inhibition has been shown to induce apoptosis in numerous cancer cells (Bahl et al. 2021; Zhang and Zhong 2014); these findings prompt the need to further explore these potential targets in selectively killing senescent cells as well. Whether through exclusive mechanisms or stronger inhibition of a key upregulated apoptosis brake in senescent cells, SR31133 is estimated to provide better mechanistic elimination of senescent cells.

A more potent senolytic fisetin analog was recently reported, containing a para‐ethoxy substitution on the B ring important for antioxidant activity (Meridew et al. 2025). However, the modification appears not to alter the antioxidant activity but instead likely improves or alters binding to specific targets. Interestingly, our fisetin analogs did not have modifications in the B ring, suggesting that the senolytic activity of fisetin can be improved through multiple types of medicinal alterations. It will be of interest to test whether the para‐ethoxy substitution on the B ring improves the activity of SR31133 and/or SR29384.

Although the in vivo pharmacodynamic effects observed across multiple tissues support systemic tissue exposure sufficient for biological activity after oral administration, formal pharmacokinetic, tissue‐distribution, and target‐engagement studies will be important next steps to define the exposure‐response relationship and guide further preclinical development of SR29384 and SR31133.

In summary, this study demonstrates the successful development of two novel flavonoid‐based senolytics, SR29384 and SR31133, through an integrative phenotypic drug discovery approach. These compounds exhibit significantly enhanced senolytic activity compared to fisetin, effectively targeting diverse senescent cell types, reducing tissue senescence, and improving selected healthspan‐associated phenotypes in mice. These promising results pave the way for preclinical and clinical studies aimed at translating SR29384 and SR31133 into viable therapeutic candidates for treating senescence‐driven age‐related diseases and promoting healthy aging. By identifying key protein targets such as PARP1 and CDK2, and elucidating their roles in DNA damage repair, apoptosis, and stress responses, this work provides valuable insights into the molecular mechanisms of senolysis. These findings not only expand the arsenal of senotherapeutics but also highlight the power of integrative approaches in bridging chemistry and biology to address complex challenges in aging and age‐related diseases.

## 4 Material and Methods

### 4.1 Experimental Design

The objective of this study was to develop novel flavonoid‐based senolytics and evaluate their translational potential for treating age‐related diseases and improving healthspan‐associated outcomes. We hypothesized that these compounds would demonstrate enhanced senolytic activity compared to fisetin, targeting key molecular pathways to selectively eliminate senescent cells. To test this hypothesis, we conducted a series of in vitro and in vivo experiments. Initial in vitro screenings assessed senolytic activity and selectivity in multiple senescent cell models. Fisetin and all flavonoid analogs were dissolved in DMSO to prepare 10 mM stock solutions for in vitro studies and were diluted into culture medium immediately before treatment; vehicle (DMSO) was matched across all conditions. In subsequent in vivo studies, the therapeutic potential of SR29384 and SR31133 was assessed using naturally aged and progeroid mouse models. For mouse studies, fisetin and lead analogs were formulated in 2% DMSO:98% vegetable oil (v/v), which provided complete solubilization. Vehicle‐treated animals received the same formulation. Evaluations focused on reductions in senescence markers (*p16* ^*Ink4a*^, *p21* ^*Cip1*^) across various tissues and improvements in healthspan metrics, including physical performance and age‐related symptoms such as tremors, kyphosis, hindlimb paralysis, gait abnormalities, dystonia, and ataxia. Mechanistic investigations were conducted using transcriptomics, molecular docking, and pathway analyses to elucidate the molecular targets of the compounds. Animals were randomly assigned to treatment or control groups. Primary endpoints included healthspan‐related outcomes, whereas secondary endpoints encompassed reductions in senescence markers and SASP factors. All animal studies were conducted in compliance with institutional guidelines and approved by the University of Minnesota Institutional Animal Care and Use Committee (IACUC).

### 4.2 Reagents

All key reagents and purchased compounds used in this study, including fluorescent probes and chemical controls, are listed below with their commercial sources and identifiers (catalog numbers and/or CAS numbers).

**Table:** 

Reagent	Source	Identifier (Catalog #; CAS)
C_12_FDG	Setareh Biotech	7188; CAS: 138777‐25‐0
Hoechst 33342	Thermo Fisher	H1399
Bafilomycin A1	Cayman Chemical	11,038; CAS: 88899‐55‐2
Formaldehyde 32%	Electron Microscopy Sciences	15,714
DPPH (2,2‐Diphenyl‐1‐picrylhydrazyl)	Sigma Aldrich	D9132
L‐Ascorbic acid	Sigma Aldrich	255,564
BCL‐XL TR‐FRET Assay Kit	BPS Bioscience	50,223
CDK2 Assay Kit	BPS Bioscience	79,599
PARP1 Colorimetric Assay Kit	BPS Bioscience	80,580
Staurosporine	Cell Signaling Technology	9953
Olaparib	Cell Signaling Technology	93,852
Fisetin	Cayman Chemical	15,246; CAS: 528‐48‐3
Quercetin	MP Biomedicals	02152003‐CF; CAS: 6151‐25‐3
Rhamnetin	Cayman Chemical	20,302; CAS: 90‐19‐7
Robinetin	INDOFINE Chemical	P‐103; CAS: 490‐31‐3
Myricetin	Cayman Chemical	10,012,600; CAS: 529‐44‐2
Quercetagetin	Cayman Chemical	18,645; CAS: 90‐18‐6
Casticin	Cayman Chemical	16,976; CAS: 479‐91‐4
Quercitrin	Cayman Chemical	19,866; CAS: 522‐12‐3
Isoquercetin	Cayman Chemical	24,926; CAS: 482‐35‐9
Myricitrin	Cayman Chemical	26,902; CAS: 17912‐87‐7
Hyperoside	Cayman Chemical	18,648; CAS: 482‐36‐0
Isoquercitroside	Cayman Chemical	20,802; CAS: 21637‐25‐2
Gossypin	BioVision	2374; CAS: 652‐78‐8
Rutin	Cayman Chemical	19,868; CAS: 250249‐75‐3
Troxerutin	Santa Cruz	sc‐203,301; CAS: 7085‐55‐4
Apigenin	Cayman Chemical	10,010,275; CAS: 520‐36‐5
Luteolin	Cayman Chemical	10,004,161; CAS: 491‐70‐3
Cirsiliol	Sigma‐Aldrich	SML0953; CAS: 34334‐69‐5
Nobiletin	Cayman Chemical	15,421; CAS: 478‐01‐3
Sinensetin	Cayman Chemical	19,751; CAS: 2306‐27‐6
PMF	INDOFINE Chemical	P20210MG; CAS: 53350‐26‐8
Luteoloside	Cayman Chemical	25,141; CAS: 5373‐11‐5
Orientin	Cayman Chemical	25,143; CAS: 28608‐75‐5
Isoorientin	Cayman Chemical	26,862; CAS: 4261‐42‐1
Homoplantaginin	Sigma‐Aldrich	SML2157; CAS: 17680‐84‐1
Lonicerin	Cayman Chemical	25,148; CAS: 25694‐72‐8
EGCG	Cayman Chemical	70,935; CAS: 989‐51‐5
GCG	Cayman Chemical	24,962; CAS: 4233‐96‐9
Dihydromyricetin	Sigma‐Aldrich	SML0295; CAS: 27200‐12‐0
Silibinin	Sigma‐Aldrich	S0417; CAS: 22888‐70‐6
Idaein	Cayman Chemical	22,264; CAS: 27661‐36‐5
Kuromanin	Cayman Chemical	16,406; CAS: 7084‐24‐4
Cyanidin	Cayman Chemical	14,803; CAS: 528‐58‐5
Peonidin	Cayman Chemical	19,754; CAS: 134‐01‐0
Theaflavin	Cayman Chemical	25,129; CAS: 4670‐05‐7
Naringenin	Cayman Chemical	14,173; CAS: 67604‐48‐2
Daidzein	Cayman Chemical	10,005,166; CAS: 486‐66‐8
(±)‐Equol	Cayman Chemical	13,184; CAS: 94105‐90‐5
THIF	Cayman Chemical	20,930; CAS: 485‐63‐2

### 4.3 Compound Synthesis and Characterization

Experimental protocols for compound synthesis and characterization were provided in Data S6.

### 4.4 Structure–Activity Relationship (SAR) Analysis and Optimization

To systematically characterize the structural requirements for senolytic activity, we employed a phenotype‐guided, iterative medicinal chemistry strategy. The SAR landscape was initially established by screening a primary library of 38 natural flavonoids in senescent *Ercc1* ^*−/−*^ and matched non‐senescent WT MEFs. This primary screen identified the shared structural features of flavone and flavonol scaffolds as a critical pharmacophoric feature for selective senolysis. Based on these, we further implemented a “Design‐Synthesize‐Test” cycle to optimize lead potency and selectivity. Structural optimization focused on three strategic regions: (1) A‐ring substitutions to enhance lipophilicity and target binding; (2) B‐ring hydroxylation patterns to maintain antioxidant/redox‐active properties; and (3) C‐ring scaffold rigidification to evaluate the impact of the C2‐C3 double bond on senolytic efficacy. All 35 synthesized analogs were evaluated in parallel across both senescent and non‐senescent cell lines.

To ensure full transparency of the SAR studies, the chemical structures and activity data for the entire series are provided in Figure 1A–C and Figure S1. All compounds were tested for full dose response to determine senolytic potency (EC_50_ in senescent cells) and the Selectivity Index (SI), calculated as the ratio of the EC_50_ in non‐senescent cells to the EC_50_ in senescent cells. Dose–response data were analyzed via non‐linear regression, and compounds failing to achieve a 50% reduction in cell viability within their maximum soluble concentration were designated as inactive (NA). This systematic approach allowed us to identify SR29384 and SR31133 as lead candidates based on their superior potency and selectivity profiles relative to the parent compound, fisetin.

### 4.5 Cells and Mice

Primary *Ercc1* ^*−/−*^ mouse embryonic fibroblasts (MEFs) and WT MEFs were isolated on embryonic day 12.5–13.5. In brief, mouse embryos were isolated from yolk sac followed by the complete removal of viscera, lung and heart if present. Embryos were then minced into fine chunks, fed with MEF medium, cultivated under 3% oxygen to reduce stress. Cells were split at 1:3 when reaching confluence. MEFs were grown at a 1:1 ratio of Dulbecco's Modification of Eagles Medium (supplemented with 4.5 g/L glucose and L‐glutamine) and Ham's F10 medium, supplemented with 10% fetal bovine serum, penicillin, streptomycin and non‐essential amino acid. To induce oxidative stress‐mediated DNA damage, *Ercc1* ^*−/−*^ MEFs were switched to 20% oxygen for three passages. WT MEFs were induced senescence by treating hydrogen peroxide H_2_O_2_ (200 μM) or etoposide (2 μM) for 24 h, followed by 5 days in normal culture media.

Human IMR90 lung fibroblasts were obtained from American Type Culture Collection (ATCC) and cultured in EMEM medium with 10% FBS and pen/strep antibiotics. To induce senescence, cells were treated with etoposide (20 μM) for 24 h, followed by 5 days in normal culture media.

Human umbilical vein endothelial cells (HUVECs) were obtained from ATCC and cultured using Endothelial Cell Growth Media plus supplement (without vascular endothelial growth factor (VEGF)) and 1% pen/strep antibiotics. The cells were experimentally treated at late passages 13 to 15.

*Ercc1* ^+/−^ and *Ercc1* ^+/*Δ*^ mice from C57BL/6J and FVB/n backgrounds were crossed to generate *Ercc1* ^−/*Δ*^ mice to prevent potential strain‐specific pathology. Aged wild‐type C57BL/6J:FVB/NJ mice were generated by crossing C57BL/6J and FVB/n inbred mice purchased from Jackson Laboratory. Mice were left to age for 2 years before being enrolled into the late life intervention study. Animal protocols used in this study were approved by the University of Minnesota Institutional Animal Care and Use Committees.

### 4.6 Healthspan Evaluation

Health assessment of *Ercc1* ^*−/Δ*^ mice was conducted twice per week to evaluate age‐related symptoms, including body weight, tremor, forelimb grip strength, kyphosis, hindlimb paralysis, gait disorder, dystonia and ataxia. Kyphosis, body condition and coat condition were used to reflect general health conditions. Ataxia, dystonia, gait disorder, and tremor were used as indicators of aging‐related neurodegeneration. All aging symptoms were scored based on a scale of 0, 0.5 and 1, except for dystonia that has a scale from 0 to 5. The sum of aging scores of each group was used to determine the overall aging conditions, with zero means no symptom presented.

### 4.7 Senotherapeutic Screening

Senescence was evaluated based on SA‐β‐gal activity using C_12_FDG staining assay. Specifically, senescent *Ercc1* ^*−/−*^ MEFs were passaged three times at 20% O_2_ to induce senescence then seeded at 3000 cells per well in black wall, clear bottom 96 well plates at least 16 h prior to treatment. Following the addition of drugs, the MEFs were incubated for 48 h at 20% O_2_. After removing the medium, cells were incubated in 100 nM Bafilomycin A1 in culture medium for 60 min to induce lysosomal alkalinization, followed by incubation with 20 μM fluorogenic substrate C_12_FDG (7188, Setareh Biotech, USA) for 2 h and counterstaining with 2 μg/mL Hoechst 33342 (H1399, Thermo Fisher Scientific, MA, USA) for 15 min. Subsequently, cells were washed with PBS and fixed in 2% paraformaldehyde for 15 min. Finally, cells were imaged with 6 fields per well using a high content fluorescent image acquisition and analysis platform Cytation 1 (BioTek, VT, USA). For SA‐β‐gal quantification, C_12_FDG‐positive cells were expressed as the percentage of total Hoechst‐positive nuclei.

For IMR90 validation and inhibitor‐probe experiments, IMR90 cells were treated with 20 μM etoposide for 24 h followed by 5 days of recovery to induce senescence, then seeded at 3000 cells per well in black‐wall, clear‐bottom 96‐well plates at least 16 h before treatment. Senescent IMR90 cells were treated with PARP1 or CDK2 inhibitors for 48 h and then processed for C_12_FDG staining as described above. Six fields were imaged per well using a Cytation 1 high‐content imaging system, and the percentage of C_12_FDG‐positive cells among Hoechst‐positive nuclei was quantified.

### 4.8 DPPH Antiradical Assay

The antioxidant activity was evaluated in microplates using the purple free radical 2,2‐diphenyl‐1‐picrylhydrazyl (DPPH•) radical assay. Methanolic solutions fisetin, SR29384, and SR31133 at different concentrations (from 0.4 to 100 μM) were tested. Methanolic solutions of L‐ascorbic acid at different concentrations were used as standards. The methanolic solution (50 μL) containing the tested compounds at different concentrations was added into 150 μL of methanolic solution of DPPH (100 μM) in 96‐well plates. The plates were incubated for 30 min under dark conditions and then measured at 517 nm using a microplate reader. The percentage inhibition of DPPH radical by the test compounds and positive control was calculated using the formula: %Inhibition = [(Abs_control—Abs_sample)/Abs_control] × 100. The IC_50_ value was calculated on the scavenging activity against DPPH radical.

### 4.9 RT‐qPCR Analysis

Total RNA was extracted from cells or snap frozen tissues using Trizol reagent (Thermo Fisher, USA). cDNA was synthesized using High‐Capacity cDNA Reverse Transcription Kit (Thermo Fisher, USA). Quantitative PCR reactions were performed with PowerUp SYBR Green Master Mix (ThermoFisher, USA). The experiments were performed according to the manufacturer's instructions. The sequences of the primers used were listed below:

**Table:** 

Gene name	Forward sequence (mouse)	Reverse sequence (mouse)
*p21* ^*Cip1*^	TCGCTGTCTTGCACTCTGGTGT	CCAATCTGCGCTTGGAGTGATAG
*p16* ^*Ink4a*^	TGTTGAGGCTAGAGAGGATCTTG	CGAATCTGCACCGTAGTTGAGC
*Tnfα*	GGTGCCTATGTCTCAGCCTCTT	GCCATAGAACTGATGAGAGGGAG
*Il6*	TACCACTTCACAAGTCGGAGGC	CTGCAAGTGCATCATCGTTGTTC
*Il1β*	TGGACCTTCCAGGATGAGGACA	GTTCATCTCGGAGCCTGTAGTG
*Mcp1*	GCTACAAGAGGATCACCAGCAG	GTCTGGACCCATTCCTTCTTGG
*Pai1*	CCTCTTCCACAAGTCTGATGGC	GCAGTTCCACAACGTCATACTCG
*Cxcl1*	TCCAGAGCTTGAAGGTGTTGCC	AACCAAGGGAGCTTCAGGGTCA
*Gapdh*	CATCACTGCCACCCAGAAGACTG	ATGCCAGTGAGCTTCCCGTTCAG

### 4.10 Pharmacokinetic Properties and *in Silico* Toxicity Screening of Compounds

Canonical SMILES of the compounds were subjected to *in silico* ADME prediction using the SWISSADME web server (Daina et al. 2017). We subsequently performed prediction algorithms for human oral toxicity parameters of the compounds using the OSIRIS Property Explorer v1.1 standalone software (https://www.organic‐chemistry.org/prog/peo) and the ProTox‐3.0 prediction tool (Banerjee et al. 2024).

### 4.11 RNA‐Seq and Transcriptomic Analysis

Non‐senescent and senescent MEF cells were treated with fisetin (5 μM), SR29384 (5 μM), or SR31133 (5 μM) for 24 h. Cells were collected and RNA samples were extracted using Trizol reagent (Thermo Fisher, USA). Samples were quantified using fluorimetry (RiboGreen assay) and RNA integrity was assessed using capillary electrophoresis. All samples had at least 500 ng mass and RNA Integrity Number (RIN) of at least 8. Library preparation was carried out using the Illumina TruSeq Stranded Total RNA Library Prep kit, followed by sequencing on the NovaSeq 6000 using 150 PE flow cell, with a sequencing depth of 20 million reads per sample. Quality control on raw sequence data for each sample was performed with FastQC. Read mapping was performed via Hisat2 (v2.1.0) using the mouse genome (GRCm38.94) as reference. Gene quantification was done via Feature Counts for raw read counts. Data have been deposited in the Gene Expression Omnibus under accession number GSE279349. Differentially expressed genes (DEGs) analysis was achieved using the R package edgeR (CPM > 0.5 cutoff). Gene Set Enrichment Analysis (GSEA) was performed using the R Clusterprofiler package (Wu et al. 2021; Yu et al. 2012). GSEA of the SenMayo panel (Saul et al. 2022) was performed using the GSEA 4.3.3 software (Mootha et al. 2003; Subramanian et al. 2005).

### 4.12 Molecular Pathway Interaction Probability Analysis

Self‐normalizing neural‐network (DSNNN) based metabolic pathway interaction probability predictions were simulated using the PathwayMap algorithm as previously described (Jimenez et al. 2019). Canonical SMILES strings for each compound were retrieved from https://pubchem.ncbi.nlm.nih.gov and submitted as inputs (listed in Data S1). Both class‐wise and individual pathway results for KEGG and Reactome interaction databases were generated and visualized using the PlayMolecule interface.

### 4.13 Structure‐Based Virtual Target Prediction and Network Construction

The canonical SMILES strings for the ligands were used as inputs for the SwissTargetPrediction algorithm (Gfeller et al. 2014). Both 2D and 3D similarity measures were used as search parameters with *H. sapiens* selected as the target database. Probability score of > 0.2 was used as statistical cutoff for identified targets and the protein identifiers were retrieved. The set of identifiers for each compound was used as node inputs in the STRING interaction database (https://string‐db.org). Results were restricted to *H. sapiens* and submitted list of proteins only. Molecular interaction modes (edges) with highest confidence score (> 0.90) were generated and used to analyze significant (FDR < 0.05) functional enrichment of biological processes (gene ontology). Subsequently, nodes and edges were imported to CytoScape v3.10.2 for visualization using the stringApp 1.7.0 add‐on. Node values were assigned using weighted k‐shell distribution via wk‐shell‐decomposition 1.1.0. Subsequently, nodes were clustered using clusterMaker2 2.0 taking undirected edges and wk‐shell values as array input with granularity parameter of 2.5. Similarly, for identifying 1st neighbor nodes of targets of interest, all shared DEGs (CPM > 0.5, |log_2_FC| > 1, FDR < 0.05) between the SR29384 versus S‐Eto and SR31133 versus S‐Eto RNA‐Seq datasets along with the target proteins were used as node inputs to construct the parent network restricted to *M. musculus* . The parent network was loaded into CytoScape, and 1st neighbor nodes of each individual target were used to construct subnetworks, targets not meeting DEG criteria were excluded. Radial diagrams of the subnetworks were generated using the yFiles Layout Algorithms 1.1.4 add‐on.

### 4.14 Ligand and Receptor Crystal Structure Acquisition and 3D Structure Preparation

The 3D ligand structures of candidate compounds and control were retrieved from https://pubchem.ncbi.nlm.nih.gov. Twenty decoy molecules per ligand were generated using the DUD‐E database (https://dude.docking.org). Prior to docking runs, we optimized the ligand structures through minimization and polar protonation (pH = 7.4). 3D crystal structures of inhibitor‐bound open conformations of the target receptors were obtained from the RCSB‐PDB database (Data S5).

The structures were optimized using Biovia Discovery Studio Modeling Environment 2024. The crystals were titrated and protonated at pH = 7.4. Co‐crystal water, non‐cofactor ligand molecules were removed, and the appropriate binding cavities were determined by the deep convolutional neural network (DCNN) based protein‐binding site prediction algorithm DeepSite (Score ≥ 0.9) (Jimenez et al. 2017), and verified with existing literature. All crystal structures were subjected to the PROCHECK algorithm for stereochemical quality assessment in order to ensure accurate docking pose predictions (https://www.ebi.ac.uk/thornton‐srv/software/PROCHECK). Estimations of the whole‐model reliability of the 3D structures were performed by evaluating the QMEAN *Z*‐scores of the PDB structures using the ProSA server (https://prosa.services.came.sbg.ac.at/prosa.php). All structures were within acceptable resolution, and *Z*‐scores for NMR and X‐ray quality crystal references (Data S5).

### 4.15 Flexible Molecular Docking Simulation

Structure‐based flexible molecular docking of the ligands into the receptor binding cavities were performed using the DockThor v2.0 platform (Santos et al. 2020). A receptor search grid of 25 Å × 25 Å × 25 Å was considered near a pre‐defined binding site of each target protein and post‐docking OPLS forcefield minimization was carried out. Binding site definitions were set using inhibitors complexed with receptors in crystal structures as templates without restraints. Monomeric subunits of the proteins were used as receptor files and grids were manually defined around the binding cavity for running the docking simulations. DMRTS method was employed for the simulation with 100,000 evaluations per run with an initial population of 750 and 25 runs per ligand. In addition to drug molecules, 20 decoy molecules per complex were used to assess the specificity of the docking protocol and the best binding decoy was used for comparisons. BAK peptide was maintained in original crystal conformation and defined as a ligand molecule to calculate binding affinity. Ligand‐receptor interactions were scored using the rDock master scoring function, and binding free energy was calculated as *Binding Free Energy (∆G) = intermolecular + ligand intramolecular + site intramolecular + external restraints* (https://rxdock.gitlab.io/documentation/devel/html/reference‐guide/scoring‐functions.html). Post‐docking visualization and ligand‐receptor interactions were analyzed using Biovia Discovery Studio Modeling Environment 2024 platform.

### 4.16 In Vitro Enzyme Activity Assays

Recombinant human CDK2/Cyclin A2 activity was measured using a luminescence‐based kinase assay (BPS Bioscience, #79599). Test compounds were incubated with CDK2/Cyclin A2, substrate peptide, and ATP according to the manufacturer's protocol, and residual ATP was quantified using the Kinase‐Glo MAX reagent. PARP1 enzymatic activity was quantified using a colorimetric assay kit (BPS Bioscience, #80580). Recombinant PARP1 was incubated with activated DNA and a biotinylated NAD^+^ substrate mixture in histone‐coated wells, and PARylation was detected using streptavidin‐HRP and a chromogenic substrate with absorbance measured at 450 nm. BCL‐xL binding activity was assessed using a homogeneous TR‐FRET assay (BPS Bioscience, #50223). Recombinant His‐tagged BCL‐XL, a biotinylated peptide ligand, terbium‐labeled anti‐His donor, and streptavidin‐conjugated acceptor were incubated with test compounds, and signals were measured at 620–665 nm following excitation at 340 nm. All assays were performed in technical triplicate, activities were normalized to vehicle‐treated controls, and IC_50_ values were calculated by nonlinear regression using a four‐parameter dose–response model using GraphPad Prism software.

### 4.17 Statistical Analysis

Statistical analyses were conducted using GraphPad Prism software. Comparisons between two groups were performed using two‐tailed Student's *t*‐tests, and comparisons among multiple groups were performed using one‐way ANOVA, as appropriate. For mouse health assessments, vehicle‐ and SR29384‐treated *Ercc1* ^−/Δ^ mice were compared at individual time points using two‐tailed Student's *t*‐tests. For mouse experiments, sample sizes are indicated in the corresponding figure legends. Data are presented as mean ± SEM for mouse studies and mean ± SD for cell‐based assays unless otherwise indicated. A value of *p* < 0.05 was considered as statistically significant, shown as *p < 0.05, **p < 0.01, ***p < 0.001 and ****p < 0.0001.

## Author Contributions

Conceptualization: L.J.Z., L.J.N., P.D.R. Methodology: L.J.Z., O.E., Q.W., Y.H., B.H., B.Z., A.N., W.X., A.M., S.J.M., L.A., R.O., T.M.K. Investigation: L.J.Z., P.D.R., R.S., C.S.‐P. Visualization: L.J.Z., R.S. Funding acquisition: L.J.Z., P.D.R. Project administration: L.J.Z., P.D.R. Supervision: L.J.Z., P.D.R. Writing – original draft: L.J.Z., R.S., C.S.‐P. Writing – review and editing: L.J.Z., P.D.R., R.S.

## Funding

Funding for this research was provided by NIH grants: R01 AG069819 (P.D.R.), P01 AG043376 (P.D.R., L.J.N.), U19 AG056278 (P.D.R., L.J.N.), R01 AG063543 (L.J.N.), P01 AG062413 (L.J.N., P.D.R.), U54 AG079754 (L.J.N., P.D.R.), U54 AG076041 (L.J.N., P.D.R.), and the Glenn Foundation for Medical Research Postdoctoral Fellowships in Aging Research (L.J.Z.).

## Conflicts of Interest

L.J.N. and P.D.R. are cofounders of Itasca Therapeutics, developing senotherapeutics for aging and age‐related diseases. L.J.Z., T.M.K., L.J.N., P.D.R. and the University of Minnesota have filed a provisional patent on the application of flavonoid analogs, including SR29384 and SR31133, as a strategy to treat age‐related diseases.

## Supporting information


**Figure S1:** Chemical structures of natural flavonoids tested in the preliminary drug screening.
**Figure S2:** Comprehensive evaluation of the pharmacological and senolytic properties of fisetin, SR29384, and SR31133. (A) BOILED‐Egg diagram and heatmap summarizing the pharmacological and physicochemical properties of fisetin, SR29384, and SR31133, including bioavailability, blood–brain barrier (BBB) permeability, solubility, and predicted toxicity. (B) Table summarizing the senolytic activity (EC_50_) and selectivity index (SI) of SR29384 and SR31133 in various senescent cell models, including *Ercc1* ^−/−^ MEFs, IMR90 fibroblasts, WT MEFs, and HUVECs. (C) RT‐qPCR analysis showing the relative mRNA expression levels of *p16* ^*Ink4a*^, *p21* ^*Cip1*^, and *Il6* in non‐senescent (NS) and senescent (S) MEF cells treated with fisetin (5 μM), SR29384 (5 μM), or SR31133 (5 μM) for 24 h. Error bars represent SD, *n* = 3. (D) Selected dose–response curves for the senolytic activity of SR29384. Error bars represent SD, *n* = 3. (E) Antioxidant activity curves of fisetin, SR29384, and SR31133, measured using the DPPH radical scavenging assay. Error bars represent SD, *n* = 2.
**Figure S3:** Acute treatment with the flavonoid analogs reduced tissue senescence in naturally aged mice. At 28 months of age, wild‐type C57BL/6J mice were administered fisetin, SR29384, or SR31133 for five consecutive days via oral gavage. Two days after the last dose, tissues were collected and analyzed. RT‐qPCR analysis of (A) lung, (B) brain, and (C) spleen showing relative mRNA expression levels of senescence markers and SASP factors across treatment groups. Error bars represent SEM; *n* = 6 mice per group.
**Figure S4:** Acute treatment with the flavonoid analogs reduced tissue senescence in naturally aged mice (continued with Figure S3). At 28 months of age, wild‐type C57BL/6J mice were administered fisetin, SR29384, or SR31133 for five consecutive days via oral gavage. Two days after the last dose, tissues were collected and analyzed. RT‐qPCR analysis of (A) gastrocnemius muscle, (B) liver, and (C) inguinal fat showing relative mRNA expression levels of senescence markers and SASP factors across treatment groups. Error bars represent SEM; *n* = 6 mice per group.
**Figure S5:** Bulk RNA‐Seq analysis of cells treated with fisetin, SR29384, and SR31133. (A) Venn diagrams showing the number of upregulated (UP) and downregulated (DOWN) DEGs that overlap between the different treatments (CPM > 0.5, |log_2_FC| > 1 and FDR < 0.05). Three independent biological replicates were analyzed per group. (B) Volcano plot comparing S‐Eto (senescent) versus NS (non‐senescent) MEFs, and S‐Eto treated with fisetin, SR29384, or SR31133 versus untreated S‐Eto MEFs. The top 20 most significantly altered genes are labeled. (C) GSEA of the SenMayo panel in S‐Eto versus NS MEFs and S‐Eto treated with fisetin, SR29384, or SR31133 versus untreated S‐Eto MEFs. (D) Bubble charts of enriched GSEA hallmark terms showing activated and suppressed pathways in S‐Eto versus NS MEFs, and S‐Eto MEFs treated with fisetin, SR29384, or SR31133 versus untreated S‐Eto MEFs.
**Figure S6:** Prediction of top drug‐pathway interactions using a deep self‐normalizing convolutional neural network model and ligand structure similarity. (A) PathwayMap KEGG class‐wise pathway interaction probability scores of compound‐pathway pairs for candidate FAs and standard senolytic compounds; pathway classes sorted by SR29384 probability scores. (B‐C) Top PathwayMap KEGG individual pathway and PathwayMap Reactome interaction probability scores of compound‐pathway pairs for candidate FAs and standard senolytic compounds; pathway classes sorted by SR29384 probability scores. (D) Ranked bubble plot of pathway (GO Biological Process) enrichment for constructed interaction network node sets for shared and exclusive SR29384 and SR31133 targets. Bubble color and size correspond to the number of observed genes in GO term, and FDR values (< 0.05).
**Figure S7:** Shared and exclusive targets among FAs predicted based on ligand structure similarity. (A) Venn diagram depicting overlaps among targets for SR29384, SR31133, fisetin, and luteolin based on SwissTargetPrediction scores. Target sets were filtered to exclude targets shared with negative control FAs (NC). (B) k‐means clustered STRING PPI maps constructed from shared and exclusive NC‐filtered target proteins for different subsets of targets. Nodes are clustered based on degree centrality.
**Figure S8:** Top drug‐pathway interactions based on ligand structure similarity for shared and exclusive targets among FAs. Ranked bubble plot of pathway (GO Biological Process) enrichment for constructed interaction network node sets of shared and exclusive targets for SR29384, SR31133, with (A) fisetin, and (B) luteolin. Bubble color and size correspond to the number of observed genes in GO term, and FDR values (< 0.05).
**Figure S9:** Expression and subnetworks of shared and exclusive molecular targets of top candidate FAs. (A) Bubble plot grid depicting changes in mRNA expression of predicted exclusive targets in etoposide‐induced senescence in MEFs, and senescent MEFs treated with SR29384 and SR31133. Genes are sorted by log_2_FC values in the SR29384 versus S‐Eto dataset. Color and sizes of bubbles correspond to a set scale for log_2_FC and FDR values; “X” symbols represent genes not detected in dataset (CPM ≤ 0.5). (B) Radial diagram of 1st neighbor node DEGs of *BCL2L1*. (C) Bubble plot grid depicting changes in mRNA expression of 1st neighbor node DEGs of *BCL2L1* in senescent MEFs treated with SR29384, SR31133, and Fisetin. (D) Radial diagram of 1st neighbor node DEGs of *CDK5R1, CBR1, GRK6, COMT, TOP1, HDAC4* and *HDAC11*. In (B) and (D), the target nodes are represented in green; hub genes within the subnetworks are in the center. Connecting edges are defined by level of interaction (probability > 0.9). (E) Ranked bubble plot depicting top 10 enriched pathways (GO Biological Processes) for upregulated and downregulated (|log_2_FC|> 1, FDR < 0.05) 1st neighbor DEGs for *BCL2L1* in senescent MEFs treated with SR29384 and SR31133. Bubble color intensity and size correspond to the number of observed genes in GO term, and FDR values (< 0.05).
**Figure S10:** SR29384 and SR31133 do not directly inhibit BCL‐xL. (A) 3D molecular model depiction of the receptor protein crystal structures used for docking simulations with surrounding native templates used for search area definition. (B) Flexible molecular docking results of BCL‐xL with candidate FAs and control compounds. Native substrate or ligand, and known inhibitor or activator drug were used along with a randomly generated structural decoy/DUD molecule. 3D and 2D interaction plots of ligands in the binding pockets with interacting pocket amino acid residues. The best‐scoring standard inhibitor was represented; ligand‐residue interaction legends are provided under the 2D plots. (C) Binding free energy scores of best‐fitting conformers for each ligand within the selected receptor binding pocket; lower binding free energies correspond to stronger ligand‐receptor interactions. Color identifiers correspond to panel (B). (D) Enzyme inhibition assays validating predicted target interactions. Recombinant BCL‐xL was incubated with increasing concentrations of SR29384 and SR31133, and residual enzymatic activity was quantified using a TR‐FRET assay. Dose–response curves were generated from normalized % activity values and fitted by nonlinear regression to determine compound potency along with an established reference inhibitor and fisetin.

**Data S1:** List of ligands and related data for PathwayMap interaction scores.

**Data S2:** Related data for gene set enrichment analyses.

**Data S3:** SwissTargetPred scores of candidate ligands.

**Data S4:** Related data for bulk RNA‐Seq results.

**Data S5:** List of target receptor crystal structures and receptor quality metrics.

**Data S6:** Chemical synthesis and characterization.

## Data Availability

All data supporting the findings of this study are available in the main text or the Supporting Information. The RNA‐seq data have been deposited in the Gene Expression Omnibus under accession number GSE279349. Materials described in this study are available upon reasonable request and may require a materials transfer agreement (MTA). Further information and requests for resources and reagents should be directed to the lead contact, Paul D. Robbins (probbins@umn.edu).

## References

[acel70736-bib-0001] Adachi, S. , H. Ito , M. Tamamori‐Adachi , et al. 2001. “Cyclin A/cdk2 Activation Is Involved in Hypoxia‐Induced Apoptosis in Cardiomyocytes.” Circulation Research 88, no. 4: 408–414. 10.1161/01.res.88.4.408. 11230108

[acel70736-bib-0002] Annunziata, C. , F. Castoldi , S. Jan , et al. 2023. “A Versatile Method for the Identification of Senolytic Compounds.” Cell Stress 7, no. 12: 105–111. 10.15698/cst2023.12.292. PMC10742873 38145234

[acel70736-bib-0003] Bahl, S. , H. Ling , N. P. N. Acharige , I. Santos‐Barriopedro , M. K. H. Pflum , and E. Seto . 2021. “EGFR Phosphorylates HDAC1 to Regulate Its Expression and Anti‐Apoptotic Function.” Cell Death & Disease 12, no. 5: 469. 10.1038/s41419-021-03697-6. PMC8113371 33976119

[acel70736-bib-0004] Baker, D. J. , B. G. Childs , M. Durik , et al. 2016. “Naturally Occurring p16(Ink4a)‐Positive Cells Shorten Healthy Lifespan.” Nature 530, no. 7589: 184–189. 10.1038/nature16932. PMC4845101 26840489

[acel70736-bib-0005] Baker, D. J. , T. Wijshake , T. Tchkonia , et al. 2011. “Clearance of p16Ink4a‐Positive Senescent Cells Delays Ageing‐Associated Disorders.” Nature 479, no. 7372: 232–236. 10.1038/nature10600. PMC3468323 22048312

[acel70736-bib-0006] Banerjee, P. , E. Kemmler , M. Dunkel , and R. Preissner . 2024. “ProTox 3.0: A Webserver for the Prediction of Toxicity of Chemicals.” Nucleic Acids Research 52, no. W1: W513–W520. 10.1093/nar/gkae303. PMC11223834 38647086

[acel70736-bib-0007] Berg, E. L. 2021. “The Future of Phenotypic Drug Discovery.” Cell Chemical Biology 28, no. 3: 424–430. 10.1016/j.chembiol.2021.01.010. 33529582

[acel70736-bib-0008] Birch, J. , and J. Gil . 2020. “Senescence and the SASP: Many Therapeutic Avenues.” Genes & Development 34, no. 23–24: 1565–1576. 10.1101/gad.343129.120. PMC7706700 33262144

[acel70736-bib-0009] Brand‐Williams, W. , M. E. Cuvelier , and C. Berset . 1995. “Use of a Free‐Radical Method to Evaluate Antioxidant Activity.” Food Science and Technology‐Lebensmittel‐Wissenschaft & Technologie 28, no. 1: 25–30. 10.1016/S0023-6438(95)80008-5.

[acel70736-bib-0010] Chen, N. , X. Chen , R. Huang , et al. 2009. “BCL‐xL Is a Target Gene Regulated by Hypoxia‐Inducible Factor‐1alpha.” Journal of Biological Chemistry 284, no. 15: 10004–10012. 10.1074/jbc.M805997200. PMC2665055 19211554

[acel70736-bib-0011] Coppe, J. P. , P. Y. Desprez , A. Krtolica , and J. Campisi . 2010. “The Senescence‐Associated Secretory Phenotype: The Dark Side of Tumor Suppression.” Annual Review of Pathology 5, no. 1: 99–118. 10.1146/annurev-pathol-121808-102144. PMC4166495 20078217

[acel70736-bib-0012] Daina, A. , O. Michielin , and V. Zoete . 2017. “SwissADME: A Free Web Tool to Evaluate Pharmacokinetics, Drug‐Likeness and Medicinal Chemistry Friendliness of Small Molecules.” Scientific Reports 7: 42717. 10.1038/srep42717. PMC5335600 28256516

[acel70736-bib-0013] Di Micco, R. , V. Krizhanovsky , D. Baker , and F. d'Adda di Fagagna . 2021. “Cellular Senescence in Ageing: From Mechanisms to Therapeutic Opportunities.” Nature Reviews Molecular Cell Biology 22, no. 2: 75–95. 10.1038/s41580-020-00314-w. PMC8344376 33328614

[acel70736-bib-0014] Dutta, C. , T. Day , N. Kopp , et al. 2012. “BCL2 Suppresses PARP1 Function and Nonapoptotic Cell Death.” Cancer Research 72, no. 16: 4193–4203. 10.1158/0008-5472.CAN-11-4204. PMC4075432 22689920

[acel70736-bib-0015] Fleury, H. , N. Malaquin , V. Tu , et al. 2019. “Exploiting Interconnected Synthetic Lethal Interactions Between PARP Inhibition and Cancer Cell Reversible Senescence.” Nature Communications 10, no. 1: 2556. 10.1038/s41467-019-10460-1. PMC6560032 31186408

[acel70736-bib-0016] Fuhrmann, D. C. , and B. Brune . 2017. “Mitochondrial Composition and Function Under the Control of Hypoxia.” Redox Biology 12: 208–215. 10.1016/j.redox.2017.02.012. PMC5333533 28259101

[acel70736-bib-0017] Gardner, L. B. , Q. Li , M. S. Park , W. M. Flanagan , G. L. Semenza , and C. V. Dang . 2001. “Hypoxia Inhibits G1/S Transition Through Regulation of p27 Expression.” Journal of Biological Chemistry 276, no. 11: 7919–7926. 10.1074/jbc.M010189200. 11112789

[acel70736-bib-0018] Gfeller, D. , A. Grosdidier , M. Wirth , A. Daina , O. Michielin , and V. Zoete . 2014. “SwissTargetPrediction: A Web Server for Target Prediction of Bioactive Small Molecules.” Nucleic Acids Research 42: W32–W38. 10.1093/nar/gku293. PMC4086140 24792161

[acel70736-bib-0019] Gorgoulis, V. , P. D. Adams , A. Alimonti , et al. 2019. “Cellular Senescence: Defining a Path Forward.” Cell 179, no. 4: 813–827. 10.1016/j.cell.2019.10.005. 31675495

[acel70736-bib-0020] Gurkar, A. U. , and L. J. Niedernhofer . 2015. “Comparison of Mice With Accelerated Aging Caused by Distinct Mechanisms.” Experimental Gerontology 68, no. Suppl C: 43–50. 10.1016/j.exger.2015.01.045. PMC4464936 25617508

[acel70736-bib-0021] Hayflick, L. , and P. S. Moorhead . 1961. “The Serial Cultivation of Human Diploid Cell Strains.” Experimental Cell Research 25: 585–621. 10.1016/0014-4827(61)90192-6. 13905658

[acel70736-bib-0022] Jiang, J. , C. B. Matranga , D. Cai , et al. 2003. “Flavopiridol‐Induced Apoptosis During S Phase Requires E2F‐1 and Inhibition of Cyclin A‐Dependent Kinase Activity.” Cancer Research 63, no. 21: 7410–7422. 14612540

[acel70736-bib-0023] Jimenez, J. , S. Doerr , G. Martinez‐Rosell , A. S. Rose , and G. De Fabritiis . 2017. “DeepSite: Protein‐Binding Site Predictor Using 3D‐Convolutional Neural Networks.” Bioinformatics 33, no. 19: 3036–3042. 10.1093/bioinformatics/btx350. 28575181

[acel70736-bib-0024] Jimenez, J. , D. Sabbadin , A. Cuzzolin , et al. 2019. “PathwayMap: Molecular Pathway Association With Self‐Normalizing Neural Networks.” Journal of Chemical Information and Modeling 59, no. 3: 1172–1181. 10.1021/acs.jcim.8b00711. 30586501

[acel70736-bib-0025] Jo, J. H. , J. J. Jo , J. M. Lee , and S. Lee . 2016. “Identification of Absolute Conversion to Geraldol From Fisetin and Pharmacokinetics in Mouse.” Journal of Chromatography. B, Analytical Technologies in the Biomedical and Life Sciences 1038: 95–100. 10.1016/j.jchromb.2016.10.034. 27810278

[acel70736-bib-0026] Jones, C. H. , and M. Dolsten . 2024. “Healthcare on the Brink: Navigating the Challenges of an Aging Society in the United States.” NPJ Aging 10, no. 1: 22. 10.1038/s41514-024-00148-2. PMC10998868 38582901

[acel70736-bib-0027] Kennedy, B. K. , S. L. Berger , A. Brunet , et al. 2014. “Geroscience: Linking Aging to Chronic Disease.” Cell 159, no. 4: 709–713. 10.1016/j.cell.2014.10.039. PMC4852871 25417146

[acel70736-bib-0028] Kirkland, J. L. , and T. Tchkonia . 2020. “Senolytic Drugs: From Discovery to Translation.” Journal of Internal Medicine 288, no. 5: 518–536. 10.1111/joim.13141. PMC7405395 32686219

[acel70736-bib-0029] Kolacz, K. , and A. Robaszkiewicz . 2024. “PARP1 at the Crossroad of Cellular Senescence and Nucleolar Processes.” Ageing Research Reviews 94: 102206. 10.1016/j.arr.2024.102206. 38278370

[acel70736-bib-0030] Lee, J. Y. , N. S. Reyes , S. Ravishankar , et al. 2024. “An In Vivo Screening Platform Identifies Senolytic Compounds That Target p16INK4a+ Fibroblasts in Lung Fibrosis.” Journal of Clinical Investigation 134, no. 9: e173371. 10.1172/JCI173371. PMC11060735 38451724

[acel70736-bib-0031] Lopez‐Otin, C. , M. A. Blasco , L. Partridge , M. Serrano , and G. Kroemer . 2013. “The Hallmarks of Aging.” Cell 153, no. 6: 1194–1217. 10.1016/j.cell.2013.05.039. PMC3836174 23746838

[acel70736-bib-0032] Lopez‐Otin, C. , M. A. Blasco , L. Partridge , M. Serrano , and G. Kroemer . 2023. “Hallmarks of Aging: An Expanding Universe.” Cell 186, no. 2: 243–278. 10.1016/j.cell.2022.11.001. 36599349

[acel70736-bib-0033] Marin, I. , M. Serrano , and F. Pietrocola . 2023. “Recent Insights Into the Crosstalk Between Senescent Cells and CD8 T Lymphocytes.” NPJ Aging 9, no. 1: 8. 10.1038/s41514-023-00105-5. PMC10073090 37015935

[acel70736-bib-0034] Martin, N. , N. Popgeorgiev , G. Ichim , and D. Bernard . 2023. “BCL‐2 Proteins in Senescence: Beyond a Simple Target for Senolysis?” Nature Reviews. Molecular Cell Biology 24, no. 8: 517–518. 10.1038/s41580-023-00594-y. 36869150

[acel70736-bib-0035] Mavrogonatou, E. , H. Pratsinis , A. Papadopoulou , N. K. Karamanos , and D. Kletsas . 2019. “Extracellular Matrix Alterations in Senescent Cells and Their Significance in Tissue Homeostasis.” Matrix Biology 75‐76: 27–42. 10.1016/j.matbio.2017.10.004. 29066153

[acel70736-bib-0036] Meridew, J. A. , J. A. Vu , D. Chow , et al. 2025. “Optimized Synthetic Flavonols Support Senescence Clearance and Lung Fibrosis Resolution.” ACS Pharmacology & Translational Science 8, no. 9: 3033–3046. 10.1021/acsptsci.5c00231. PMC12441843 40969882

[acel70736-bib-0037] Moffat, J. G. , F. Vincent , J. A. Lee , J. Eder , and M. Prunotto . 2017. “Opportunities and Challenges in Phenotypic Drug Discovery: An Industry Perspective.” Nature Reviews. Drug Discovery 16, no. 8: 531–543. 10.1038/nrd.2017.111. 28685762

[acel70736-bib-0038] Mootha, V. K. , C. M. Lindgren , K. F. Eriksson , et al. 2003. “PGC‐1alpha‐Responsive Genes Involved in Oxidative Phosphorylation Are Coordinately Downregulated in Human Diabetes.” Nature Genetics 34, no. 3: 267–273. 10.1038/ng1180. 12808457

[acel70736-bib-0039] Nehme, J. , L. Mesilmany , V. Farfariello , et al. 2023. PARP1 Inhibition Mediates a Switch From Necrosis to Senescence That Favors Repair From Acute Oxidative Injury. Research Square. 10.21203/rs.3.rs-2672861/v1.

[acel70736-bib-0040] Niccoli, T. , and L. Partridge . 2012. “Ageing as a Risk Factor for Disease.” Current Biology 22, no. 17: R741–R752. 10.1016/j.cub.2012.07.024. 22975005

[acel70736-bib-0041] Prasnikar, E. , J. Borisek , and A. Perdih . 2021. “Senescent Cells as Promising Targets to Tackle Age‐Related Diseases.” Ageing Research Reviews 66: 101251. 10.1016/j.arr.2020.101251. 33385543

[acel70736-bib-0042] Proshkina, E. , L. Koval , E. Platonova , et al. 2024. “Polyphenols as Potential Geroprotectors.” Antioxidants & Redox Signaling 40, no. 7–9: 564–593. 10.1089/ars.2023.0247. 38251662

[acel70736-bib-0043] Rahmani, A. H. , A. Almatroudi , K. S. Allemailem , A. A. Khan , and S. A. Almatroodi . 2022. “The Potential Role of Fisetin, a Flavonoid in Cancer Prevention and Treatment.” Molecules 27, no. 24: 9009. 10.3390/molecules27249009. PMC9782831 36558146

[acel70736-bib-0044] Rauf, A. , T. Abu‐Izneid , M. Imran , et al. 2023. “Therapeutic Potential and Molecular Mechanisms of the Multitargeted Flavonoid Fisetin.” Current Topics in Medicinal Chemistry 23, no. 21: 2075–2096. 10.2174/1568026623666230710162217. 37431899

[acel70736-bib-0045] Ravula, A. R. , S. B. Teegala , S. Kalakotla , J. P. Pasangulapati , V. Perumal , and H. K. Boyina . 2021. “Fisetin, Potential Flavonoid With Multifarious Targets for Treating Neurological Disorders: An Updated Review.” European Journal of Pharmacology 910: 174492. 10.1016/j.ejphar.2021.174492. 34516952

[acel70736-bib-0046] Rello‐Varona, S. , M. Fuentes‐Guirado , R. Lopez‐Alemany , et al. 2019. “Bcl‐x(L) Inhibition Enhances Dinaciclib‐Induced Cell Death in Soft‐Tissue Sarcomas.” Scientific Reports 9, no. 1: 3816. 10.1038/s41598-019-40106-7. PMC6405759 30846724

[acel70736-bib-0047] Robbins, P. D. , D. Jurk , S. Khosla , et al. 2021. “Senolytic Drugs: Reducing Senescent Cell Viability to Extend Health Span.” Annual Review of Pharmacology and Toxicology 61, no. 1: 779–803. 10.1146/annurev-pharmtox-050120-105018. PMC7790861 32997601

[acel70736-bib-0048] Samuelsson, M. K. , A. Pazirandeh , and S. Okret . 2002. “A Pro‐Apoptotic Effect of the CDK Inhibitor p57(Kip2) on Staurosporine‐Induced Apoptosis in HeLa Cells.” Biochemical and Biophysical Research Communications 296, no. 3: 702–709. 10.1016/s0006-291x(02)00912-9. 12176039

[acel70736-bib-0049] Santos, K. B. , I. A. Guedes , A. L. M. Karl , and L. E. Dardenne . 2020. “Highly Flexible Ligand Docking: Benchmarking of the DockThor Program on the LEADS‐PEP Protein‐Peptide Data Set.” Journal of Chemical Information and Modeling 60, no. 2: 667–683. 10.1021/acs.jcim.9b00905. 31922754

[acel70736-bib-0050] Saul, D. , R. L. Kosinsky , E. J. Atkinson , et al. 2022. “A New Gene Set Identifies Senescent Cells and Predicts Senescence‐Associated Pathways Across Tissues.” Nature Communications 13, no. 1: 4827. 10.1038/s41467-022-32552-1. PMC9381717 35974106

[acel70736-bib-0051] Seguin, J. , L. Brulle , R. Boyer , et al. 2013. “Liposomal Encapsulation of the Natural Flavonoid Fisetin Improves Bioavailability and Antitumor Efficacy.” International Journal of Pharmaceutics 444, no. 1–2: 146–154. 10.1016/j.ijpharm.2013.01.050. 23380621

[acel70736-bib-0052] Softah, A. , M. R. Alotaibi , A. R. Alhoshani , et al. 2023. “The Combination of Radiation With PARP Inhibition Enhances Senescence and Sensitivity to the Senolytic, Navitoclax, in Triple Negative Breast Tumor Cells.” Biomedicines 11, no. 11: 3066. 10.3390/biomedicines11113066. PMC10669784 38002066

[acel70736-bib-0053] Subramanian, A. , P. Tamayo , V. K. Mootha , et al. 2005. “Gene Set Enrichment Analysis: A Knowledge‐Based Approach for Interpreting Genome‐Wide Expression Profiles.” Proceedings of the National Academy of Sciences of the United States of America 102, no. 43: 15545–15550. 10.1073/pnas.0506580102. PMC1239896 16199517

[acel70736-bib-0054] Sundarraj, K. , A. Raghunath , and E. Perumal . 2018. “A Review on the Chemotherapeutic Potential of Fisetin: In Vitro Evidences.” Biomedicine & Pharmacotherapy 97, no. Suppl C: 928–940. 10.1016/j.biopha.2017.10.164. 29136771

[acel70736-bib-0055] Tse, C. , A. R. Shoemaker , J. Adickes , et al. 2008. “ABT‐263: A Potent and Orally Bioavailable Bcl‐2 Family Inhibitor.” Cancer Research 68, no. 9: 3421–3428. 10.1158/0008-5472.CAN-07-5836. 18451170

[acel70736-bib-0056] van de Waterbeemd, H. , G. Camenisch , G. Folkers , J. R. Chretien , and O. A. Raevsky . 1998. “Estimation of Blood‐Brain Barrier Crossing of Drugs Using Molecular Size and Shape, and H‐Bonding Descriptors.” Journal of Drug Targeting 6, no. 2: 151–165. 10.3109/10611869808997889. 9886238

[acel70736-bib-0057] Vincent, F. , A. Nueda , J. Lee , M. Schenone , M. Prunotto , and M. Mercola . 2022. “Phenotypic Drug Discovery: Recent Successes, Lessons Learned and New Directions.” Nature Reviews. Drug Discovery 21, no. 12: 899–914. 10.1038/s41573-022-00472-w. PMC9708951 35637317

[acel70736-bib-0058] Wang, B. , L. Wang , N. S. Gasek , et al. 2021. “An Inducible p21‐Cre Mouse Model to Monitor and Manipulate p21‐Highly‐Expressing Senescent Cells In Vivo.” Nature Aging 1, no. 10: 962–973. 10.1038/s43587-021-00107-6. PMC8746571 35024619

[acel70736-bib-0059] Wiley, C. D. , and J. Campisi . 2021. “The Metabolic Roots of Senescence: Mechanisms and Opportunities for Intervention.” Nature Metabolism 3, no. 10: 1290–1301. 10.1038/s42255-021-00483-8. PMC8889622 34663974

[acel70736-bib-0060] Wu, T. , E. Hu , S. Xu , et al. 2021. “clusterProfiler 4.0: A Universal Enrichment Tool for Interpreting Omics Data.” Innovation (Cambridge, Mass.) 2, no. 3: 100141. 10.1016/j.xinn.2021.100141. PMC8454663 34557778

[acel70736-bib-0061] Xie, T. , J. Yuan , L. Mei , P. Li , and R. Pan . 2022. “Luteolin Suppresses TNF‐Alpha‐Induced Inflammatory Injury and Senescence of Nucleus Pulposus Cells via the Sirt6/NF‐kappaB Pathway.” Experimental and Therapeutic Medicine 24, no. 1: 469. 10.3892/etm.2022.11396. PMC9204575 35747154

[acel70736-bib-0062] Yan, J. , C. L. Sun , S. Shin , M. Van Gilst , and C. M. Crowder . 2021. “Effect of the Mitochondrial Unfolded Protein Response on Hypoxic Death and Mitochondrial Protein Aggregation.” Cell Death & Disease 12, no. 7: 711. 10.1038/s41419-021-03979-z. PMC8282665 34267182

[acel70736-bib-0063] Yousefzadeh, M. J. , J. Zhao , C. Bukata , et al. 2020. “Tissue Specificity of Senescent Cell Accumulation During Physiologic and Accelerated Aging of Mice.” Aging Cell 19, no. 3: e13094. 10.1111/acel.13094. PMC7059165 31981461

[acel70736-bib-0064] Yousefzadeh, M. J. , Y. Zhu , S. J. McGowan , et al. 2018. “Fisetin Is a Senotherapeutic That Extends Health and Lifespan.” eBioMedicine 36: 18–28. 10.1016/j.ebiom.2018.09.015. PMC6197652 30279143

[acel70736-bib-0065] Yu, C. Y. , T. K. Yeung , W. K. Fu , and R. Y. C. Poon . 2024. “BCL‐XL Regulates the Timing of Mitotic Apoptosis Independently of BCL2 and MCL1 Compensation.” Cell Death & Disease 15, no. 1: 2. 10.1038/s41419-023-06404-9. PMC10764939 38172496

[acel70736-bib-0066] Yu, G. , L. G. Wang , Y. Han , and Q. Y. He . 2012. “clusterProfiler: An R Package for Comparing Biological Themes Among Gene Clusters.” OMICS 16, no. 5: 284–287. 10.1089/omi.2011.0118. PMC3339379 22455463

[acel70736-bib-0067] Zhang, J. , and Q. Zhong . 2014. “Histone Deacetylase Inhibitors and Cell Death.” Cellular and Molecular Life Sciences 71, no. 20: 3885–3901. 10.1007/s00018-014-1656-6. PMC4414051 24898083

[acel70736-bib-0068] Zhang, L. , L. E. Pitcher , V. Prahalad , L. J. Niedernhofer , and P. D. Robbins . 2021. “Recent Advances in the Discovery of Senolytics.” Mechanisms of Ageing and Development 200: 111587. 10.1016/j.mad.2021.111587. PMC8687661 34656616

[acel70736-bib-0069] Zhang, L. , L. E. Pitcher , V. Prahalad , L. J. Niedernhofer , and P. D. Robbins . 2023. “Targeting Cellular Senescence With Senotherapeutics: Senolytics and Senomorphics.” FEBS Journal 290, no. 5: 1362–1383. 10.1111/febs.16350. 35015337

[acel70736-bib-0070] Zhang, L. , L. E. Pitcher , M. J. Yousefzadeh , L. J. Niedernhofer , P. D. Robbins , and Y. Zhu . 2022. “Cellular Senescence: A Key Therapeutic Target in Aging and Diseases.” Journal of Clinical Investigation 132, no. 15: e158450. 10.1172/JCI158450. PMC9337830 35912854

[acel70736-bib-0071] Zhu, H. , M. Wei , J. Xu , et al. 2020. “PARP Inhibitors in Pancreatic Cancer: Molecular Mechanisms and Clinical Applications.” Molecular Cancer 19, no. 1: 49. 10.1186/s12943-020-01167-9. PMC7053129 32122376

[acel70736-bib-0072] Zhu, R. Z. , B. S. Li , S. S. Gao , J. H. Seo , and B. M. Choi . 2021. “Luteolin Inhibits H(2)O(2)‐Induced Cellular Senescence via Modulation of SIRT1 and p53.” Korean Journal of Physiology & Pharmacology 25, no. 4: 297–305. 10.4196/kjpp.2021.25.4.297. PMC8255127 34187948

[acel70736-bib-0073] Zhu, T. , J. Y. Zheng , L. L. Huang , Y. H. Wang , D. F. Yao , and H. B. Dai . 2023. “Human PARP1 Substrates and Regulators of Its Catalytic Activity: An Updated Overview.” Frontiers in Pharmacology 14: 1137151. 10.3389/fphar.2023.1137151. PMC9995695 36909172

[acel70736-bib-0074] Zhu, Y. , E. J. Doornebal , T. Pirtskhalava , et al. 2017. “New Agents That Target Senescent Cells: The Flavone, Fisetin, and the BCL‐X(L) Inhibitors, A1331852 and A1155463.” Aging (Albany NY) 9, no. 3: 955–963. 10.18632/aging.101202. PMC5391241 28273655

[acel70736-bib-0075] Zumerle, S. , M. Sarill , M. Saponaro , et al. 2024. “Targeting Senescence Induced by Age or Chemotherapy With a Polyphenol‐Rich Natural Extract Improves Longevity and Healthspan in Mice.” Nature Aging 4, no. 9: 1231–1248. 10.1038/s43587-024-00663-7. PMC11408255 38951692

